# Carbohydrate Metabolic Compensation Coupled to High Tolerance to Oxidative Stress in Ticks

**DOI:** 10.1038/s41598-019-41036-0

**Published:** 2019-03-18

**Authors:** Bárbara Della Noce, Marcelle Vianna de Carvalho Uhl, Josias Machado, Camila Fernanda Waltero, Leonardo Araujo de Abreu, Renato Martins da Silva, Rodrigo Nunes da Fonseca, Cintia Monteiro de Barros, Gabriela Sabadin, Satoru Konnai, Itabajara da Silva Vaz, Kazuhiko Ohashi, Carlos Logullo

**Affiliations:** 1 Laboratório Integrado de Bioquímica Hatisaburo Masuda and Laboratório Integrado de Morfologia, NUPEM-UFRJ, Macaé, RJ Brazil; 2 Centro de Biotecnologia and Faculdade de Veterinária – UFRGS, Porto Alegre, RS Brazil; 3 Laboratory of Infectious Diseases, Hokkaido University, Sapporo, 060-0818 Japan; 4 Instituto de Bioquímica Médica Leopoldo de Meis, Universidade Federal do Rio de Janeiro, Rio de Janeiro, RJ Brazil; 5 Instituto Nacional de Ciência e Tecnologia em Entomologia Molecular, Rio de Janeiro, RJ Brazil

## Abstract

Reactive oxygen species (ROS) are natural byproducts of metabolism that have toxic effects well documented in mammals. In hematophagous arthropods, however, these processes are not largely understood. Here, we describe that *Rhipicephalus microplus* ticks and embryonic cell line (BME26) employ an adaptive metabolic compensation mechanism that confers tolerance to hydrogen peroxide (H_2_O_2_) at concentrations too high for others organisms. Tick survival and reproduction are not affected by H_2_O_2_ exposure, while BME26 cells morphology was only mildly altered by the treatment. Furthermore, H_2_O_2_-tolerant BME26 cells maintained their proliferative capacity unchanged. We evaluated several genes involved in gluconeogenesis, glycolysis, and pentose phosphate pathway, major pathways for carbohydrate catabolism and anabolism, describing a metabolic mechanism that explains such tolerance. Genetic and catalytic control of the genes and enzymes associated with these pathways are modulated by glucose uptake and energy resource availability. Transient increase in ROS levels, oxygen consumption, and ROS-scavenger enzymes, as well as decreased mitochondrial superoxide levels, were indicative of cell adaptation to high H_2_O_2_ exposure, and suggested a tolerance strategy developed by BME26 cells to cope with oxidative stress. Moreover, NADPH levels increased upon H_2_O_2_ challenge, and this phenomenon was sustained mainly by G6PDH activity. Interestingly, G6PDH knockdown in BME26 cells did not impair H_2_O_2_ tolerance, but generated an increase in NADP-ICDH transcription. In agreement with the hypothesis of a compensatory NADPH production in these cells, NADP-ICDH knockdown increased G6PDH relative transcript level. The present study unveils the first metabolic evidence of an adaptive mechanism to cope with high H_2_O_2_ exposure and maintain redox balance in ticks.

## Introduction

Among the diverse range of reactive oxygen species (ROS), hydrogen peroxide (H_2_O_2_) seems to be the most important signaling compound, as suggested by studies in mammalian cells, where it is reported to be continuously produced in a steady-state concentration between 10^−7^ M and 10^−9^ M^1–4^. Energy metabolism mechanisms work chiefly to supply the organism’s energetic demand, but also to maintain physiological homeostasis and to prevent oxidative damage caused by ROS generated as byproducts^5–7^. For instance, glucose metabolism includes both ROS generation and scavenging processes^6^. The activity of the first glycolytic enzyme, hexokinase, depends on ATP generated by mitochondrial ATP-synthase, thus supplying ADP to sustain the flow of electrons through oxidative phosphorylation, which in turn prevents free electrons from reacting with oxygen and the consequent generation of ROS^8,9^.

Recently, arthropod cell lines were established as models to study several biological processes, including metabolism, signaling, vector-pathogen interactions, and oxidative stress^10–14^. Understanding the biochemical basis of ROS homeostasis in these cell lines might provide new molecular targets for the control of invertebrate parasites and disease vectors^15^. In the interest of understanding how hematophagous arthropods cope with oxidative stress caused by the high amounts of heme ingested during blood feeding, several biochemical studies have been performed in organisms such as *Rhodinus prolixus*^16^, *Aedes aegypti*^17^ and *Rhipicephalus microplus*^18^. Some of the mechanisms described include: free heme reduction by formation of insoluble aggregates, heme complexation with ligand proteins, and plain heme degradation^19,20^. On the other hand, despite the large number of publications that involve oxidative response to blood feeding in ticks, the higher hydrogen peroxide tolerance observed in these organisms is poorly documented, as are the metabolic events that lead to this high tolerance. The Pentose Phosphate Pathway (PPP) is an alternative oxidative route for glucose that also provides glycolytic intermediates and nucleotide precursors, CO_2_ and reduced nicotinamide adenine dinucleotide phosphate (NADPH)^21–23^. Intracellular NADPH provides reducing potential for biosynthetic reactions and protection against oxidative compounds^24^, and may also be supplied by isocitrate dehydrogenase and malic enzyme^25,26^.

In the present work, *R. microplus* cattle tick demonstrated a remarkable resistance to high H_2_O_2_ concentrations, with unaffected survival and reproduction rates. We have used the BME26 tick cell line challenged with H_2_O_2_ to investigate a so-far undisclosed adaptive strategy that reduces ROS levels by regulating both transcription and activity of enzymes associated with aerobic and anaerobic carbohydrate metabolism and NADPH production. Such metabolic compensation makes ticks remarkably tolerant to oxidative stress.

## Results

### Ticks showed high tolerance to H_2_O_2_ exposure

**Figure 1:**
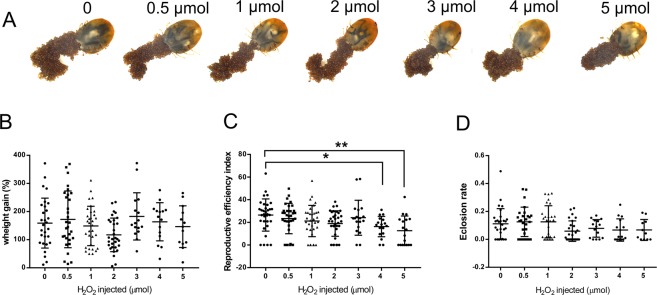
*R. microplus* tick endures H_2_O_2_ overexposure. H_2_O_2_ tolerance in ticks was evaluated measuring biological parameters after H_2_O_2_ injection in partially fed females. (**A**) Representative images of ticks after completed oviposition. Ticks were collected from groups injected with increasing amounts of H_2_O_2_ (0.5–5 μmol). (**B**) Tick weight gain was used to determine blood feeding capacity; (**C**) Tick reproductive efficiency index was used to determine the oviposition capacity; and (**D**) Eclosion rate, after H_2_O_2_ injection followed by completed engorgement by capillary tube blood feeding. Groups of 33 females were used for treatment at 0, 0.5, 1 and 2 µmol, and 17 females for each group treated at 3, 4 and 5 µmol. Data was verified for normal distribution using D’Agostino & Pearson normality test, statistical significance was assessed by Ordinary one-way ANOVA, being *p 0,0482, **p 0,0022.

Overexposure of biological systems to H_2_O_2_ is related to deleterious effects on cells, tissues and organisms^27–30^. In order to investigate H_2_O_2_ susceptibility of ticks *in vivo* we injected 1 µL of H_2_O_2_ in partially fed females at concentrations ranging from 0.5 to 5 M (~2,5–25 mM final concentration) and analyzed the immediate impacts and after effects on tick blood meal and reproduction (Fig. 1). We observed that females were able to survive H_2_O_2_ injections of up to 5 µmol. H_2_O_2_ injection caused immediate strong reaction inside the tick, followed by an overflow of the internal contents, especially at amounts greater than 1 µmol (Supplementary Video 1).

Surprisingly, treated ticks recovered from this injury (Fig. 1A) and were able to feed as successfully as control ticks (Fig. 1B), demonstrating their high H_2_O_2_ tolerance and ability to counteract H_2_O_2_-induced damage. Treated ticks were also able to lay eggs and these eggs were viable (Fig. 1C,D). Among all evaluated biological parameters, only reproductive efficiency index was mildly affected at higher concentrations (Fig. 1C). This is an exceptional survival capacity, unparalleled in other models. H_2_O_2_ exposure at millimolar concentrations induced significant mortality in *Drosophila melanogaster*^31^ and *Caenorhabditis elegans*^32^.

### Tick embryonic BME26 cells showed high tolerance to H_2_O_2_ exposure and mildly altered cell morphology

**Figure 2:**
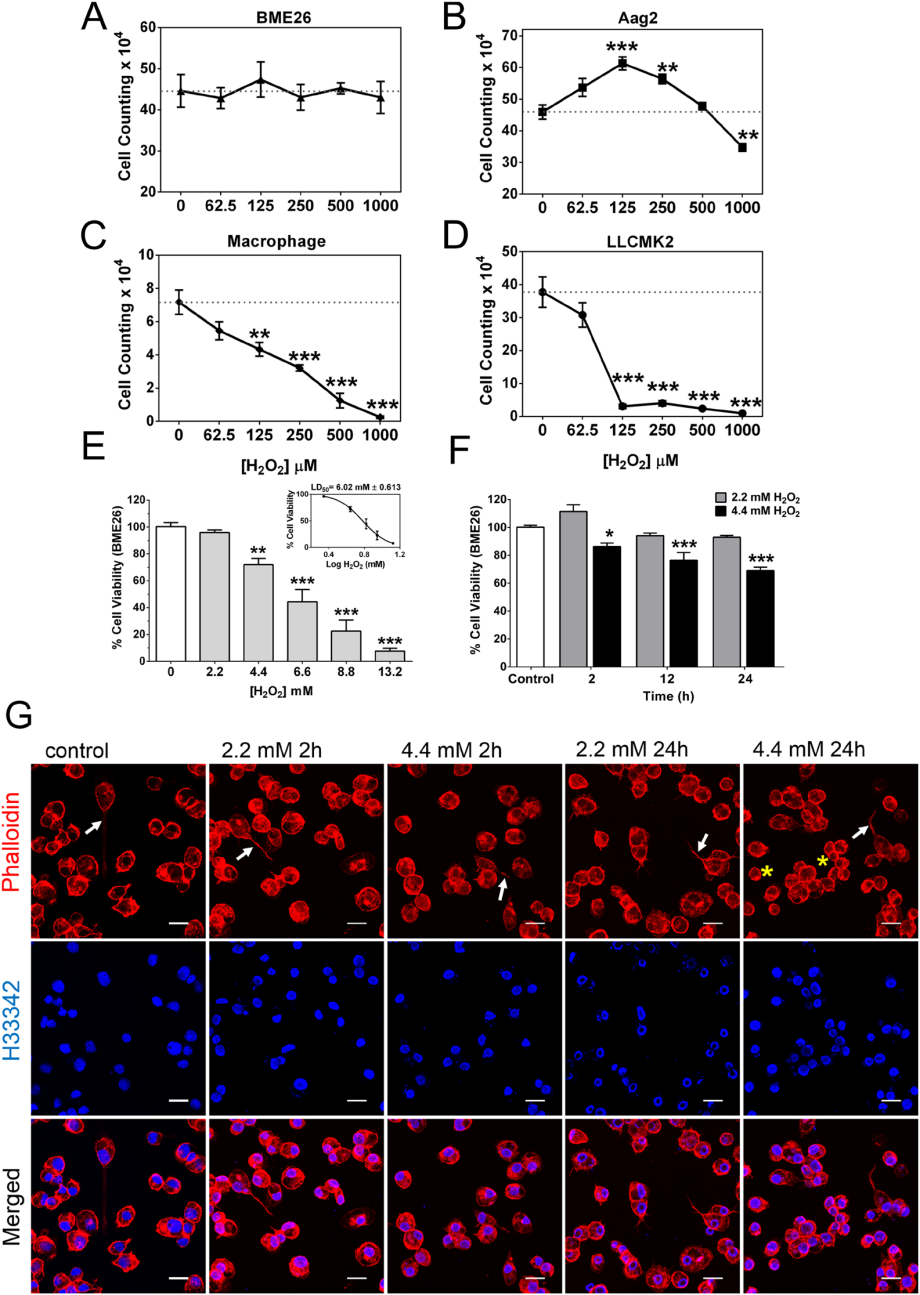
BME26 tick cells tolerate H_2_O_2_ overexposure compared to other mammalian cell lines. (**A**–**D**) Comparison of cell viability was determined using a Neubauer hemocytometer with trypan blue exclusion technique, in four different cell types 24 h after challenge with increasing H_2_O_2_ concentrations (from 62.5 μM to 1000 μM H_2_O_2_). (**A**) Embryonic *Rhipicephalus microplus* BME26 cell line; (**B**) embryonic *Aedes aegypti* Aag2 cell line; (**C**) BLACK6 mouse macrophage primary culture cells; (**D**) Rhesus monkey kidney epithelial LLCMK2 cell line. The dotted line (100%) represents untreated control (absence of H_2_O_2_). (**E**) BME26 cells viability was measured by MTT assay 24 h after H_2_O_2_ addition at increasing concentrations ranging from 2.2 mM to 13.2 mM. Insert shows LD_50_ at approximately 6.02 mM. (**F**) BME26 cells viability was assessed by MTT assay 2 h, 12 h and 24 h after H_2_O_2_ addition at 2.2 mM (non-lethal) and 4.4 mM (~LD_25_) concentrations. (**G**) BME26 cells morphology was observed under a confocal laser scanning microscope, Zeiss LSM 710. Cytoskeletal architecture (red) and nuclei (blue) were observed using Phalloidin-Texas Red and Hoechst 33342 staining, respectively. White arrows show the filopodia and yellow asterisks show cells with a rounded shape. Scale Bar: 20 µm. The experiments were performed with three independent biological samples in three experimental replicates each, *p < 0.05, **p < 0.01, ***p < 0.001, compared to control in Tukey’s multiple comparisons test.

To investigate the metabolic mechanisms related to H_2_O_2_ tolerance, we used BME26 tick embryonic cells challenged with direct addition of H_2_O_2_^33^. The effect of oxidative conditions on cell survival was compared among different cell types. In general, at concentrations ranging from 62.5–1000 µM H_2_O_2_, arthropod embryonic cells (BME26 from *R. microplus* and Aag2 from *A. aegypti*) were more tolerant than mouse macrophages (primary culture) and Rhesus monkey kidney epithelial cells (LLCMK2) (Fig. 2A–D). Similarly, cell viability assay showed that *Drosophila* Schneider 2 (S2) cell line was able to tolerate up to 1 mM H_2_O_2_, and exhibited a 20% decrease in cell viability when exposed to 2 mM (2000 µM) H_2_O_2_ (Supplementary Fig. S1B). In both mammalian cell types, H_2_O_2_ induced cytotoxic effects leading to reduced cellular viability; LD_50_ values ranged between 125–250 μM H_2_O_2_ after 24 h of treatment, in agreement with previous findings (Fig. 2C,D)^34,35^. In contrast, the mosquito embryonic cell line Aag2 exhibited cell proliferation at concentrations ranging from 125 to 250 µM H_2_O_2_, with up to 30% increase in viable cell number. Only at the highest tested concentration (1000 µM) H_2_O_2_ reduced by 20% the number of viable Aag2 cells (Fig. 2B). BME26 cells were unaffected by H_2_O_2_ at concentrations from 62.5 to 1000 μM, with number of viable cells similar to the untreated control, as determined by Trypan blue exclusion (Fig. 2A), as well as by reduction of 3-(4,5-dimethylthiazol-2-yl)-2,5-diphenyltetrazolium bromide (MTT) to its insoluble formazan (Supplementary Fig. S1A).

Then, to determine the lethal dose (LD), BME26 cell viability was evaluated 24 h after H_2_O_2_ treatment at millimolar concentrations (2.2 mM to 13.2 mM). Viability was not significantly affected at 2.2 mM H_2_O_2_, but a dose-dependent reduction in cell viability was observed at concentrations between 4.4 mM and 13.2 mM. The highest concentration caused 90% reduction in the number of viable cells (Fig. 2E), and the LD_50_ 24 h after H_2_O_2_ challenge was determined at 6.02 ± 0.613 mM (Fig. 2E insert).

Further investigation was performed to understand BME26 cells tolerance, using H_2_O_2_ concentrations of 2.2 mM (non-lethal) and 4.4 mM (LD_25_). Treatment with 2.2 mM H_2_O_2_ did not cause significant alterations in cell viability over time from 2 h up to 24 h (Fig. 2F and Supplementary Fig. S1C, gray bars). At 4.4 mM H_2_O_2_, cell viability was mildly but significantly affected in a time-dependent manner (Fig. 2F and Supplementary Fig. S1C, black bars), an effect accompanied by morphological changes (Fig. 2G and Supplementary Fig. S1D).

BME26 cells were previously characterized as a morphologically heterogeneous cell type, similar to other arthropod lines, presenting different sizes of cells and nuclei^36^. Two major cell morphologies are usually observed in a culture of tick cells, one with a fusiform appearance with several cellular filopodia, and another presenting rounded and larger format with a large number of vesicles scattered through the cytoplasm. These two morphologies characterize a tick cell culture under optimal conditions^36,37^. Using phalloidin stain after treatment with H_2_O_2_, a reduction in number of cell events with fusiform appearance and filopodia (white arrows) was observed, with a higher proportion of rounded small cells (yellow asterisks, Fig. 2G). This phenomenon was more evident 24 h after challenge at 4.4 mM H_2_O_2_ compared to shorter exposure time or lower dose treatment, which was also corroborated by cytochemical staining with Quick-Panoptic (Supplementary Fig. S1D). The morphological alteration of cellular retraction can be associated with the reduction in cellular viability^38^, or might indicate a possible trans-differentiation phenomenon among the different cell formats, leading to filopodia reduction^39^.

### H_2_O_2_ challenge did not affect the proliferative capacity of BME26 cells

**Figure 3:**
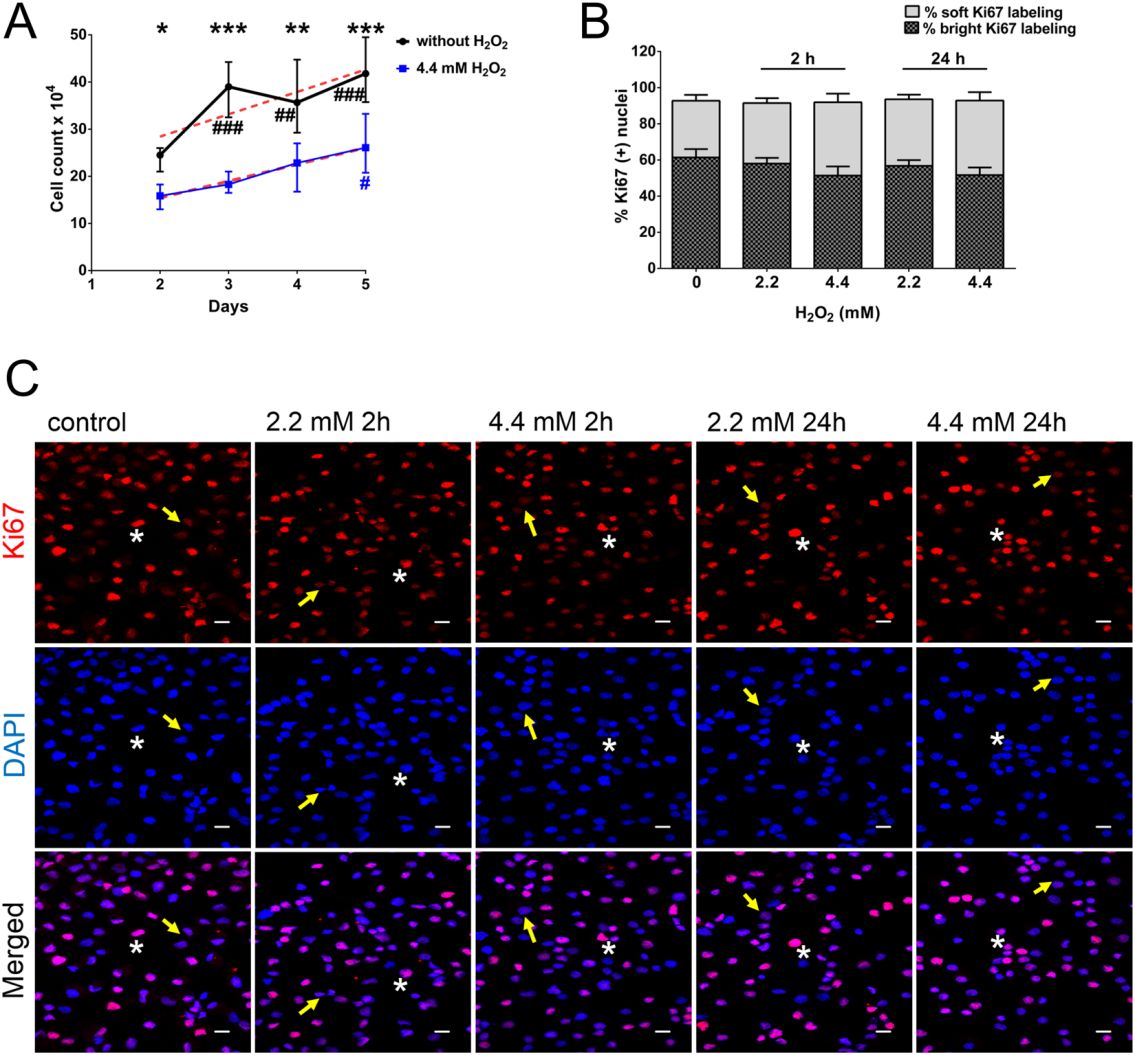
Proliferative capacity was unchanged in BME26 cells after H_2_O_2_ challenge. (**A**) Cell viability was assessed by cell counting with trypan blue dye exclusion 2, 3, 4 and 5 days after 4.4 mM H_2_O_2_ addition. Dotted red line represents slope of the cell growth line shown in control (4.738 ± 1.27) and treated (3.545 ± 0.68) conditions. ^∗^p < 0,05, ^∗∗^p < 0,01, ^∗∗∗^p < 0,0001, comparing 4.4 mM treatment to the control conditions; and ^#^p < 0,05; ^##^p < 0,01; ^###^p < 0,0001, comparing the same treatment over time, in Tukey’s multiple comparisons test. (**B**) Count of nuclei labeled with Ki67 in two categories (soft and bright) of red nuclei were separately normalized to total nuclei (DAPI stained) as 100%. (**C**) Ki67 immunolocalization and DAPI nuclear staining in BME26 cells were visualized under laser scanning confocal microscope LSM 710, Zeiss. Yellow arrows indicate Ki67 soft labeling and white asterisks indicates Ki67 bright labeling Scale Bar: 10 µm. The experiments were performed with three independent biological samples in three experimental replicates each.

Because cell retraction may be associated with reduction in cellular viability, we evaluated the viable cell counts during 5 days after 4.4 mM H_2_O_2_ treatment (Fig. 3A). Interestingly, an increased number of viable cells were observed comparing the 2^nd^ and the 5^th^ day after treatment, with approximately 40% more cells at the end of this period (blue line, Fig. 3A). Cell growth under control or treated conditions showed a similar slope (4.738 ± 1.27 and 3.545 ± 0.68, respectively; Fig. 3A, dotted red lines). To confirm that the cells retained the same proliferative capacity even after H_2_O_2_ treatment, proliferation was analyzed by fluorescence-based immunolocalization of Ki67, a cell proliferation marker. Quantitative image analysis was performed using Cell Counter tool on ImageJ. Among the nuclei labeled with anti-Ki67, different degrees of intensity were identified corresponding to variation in Ki67 protein levels. Two categories (soft and bright) of red nuclei were separately normalized to total nuclei (DAPI stained) as 100% (Fig. 3B). The results show that H_2_O_2_ did not alter the proliferative capacity of BME26 cells (Fig. 3B,C).

For the first time we have observed that BME26 cells can tolerate millimolar H_2_O_2_ concentrations. At the millimolar range we chose a non-lethal dose and LD_25_ concentration for further experiments. We evaluated cell survival and proliferation, and adaptability in this extremely oxidative extracellular environment by analyzing intracellular ROS profile, gene transcription, and activity of H_2_O_2_-scavenger enzymes.

### H_2_O_2_ challenge transiently altered levels of ROS, oxygen consumption, ROS-scavenger enzymes, and mitochondrial superoxide in BME26 cells, pointing to a cellular adaptation tolerance mechanism

**Figure 4:**
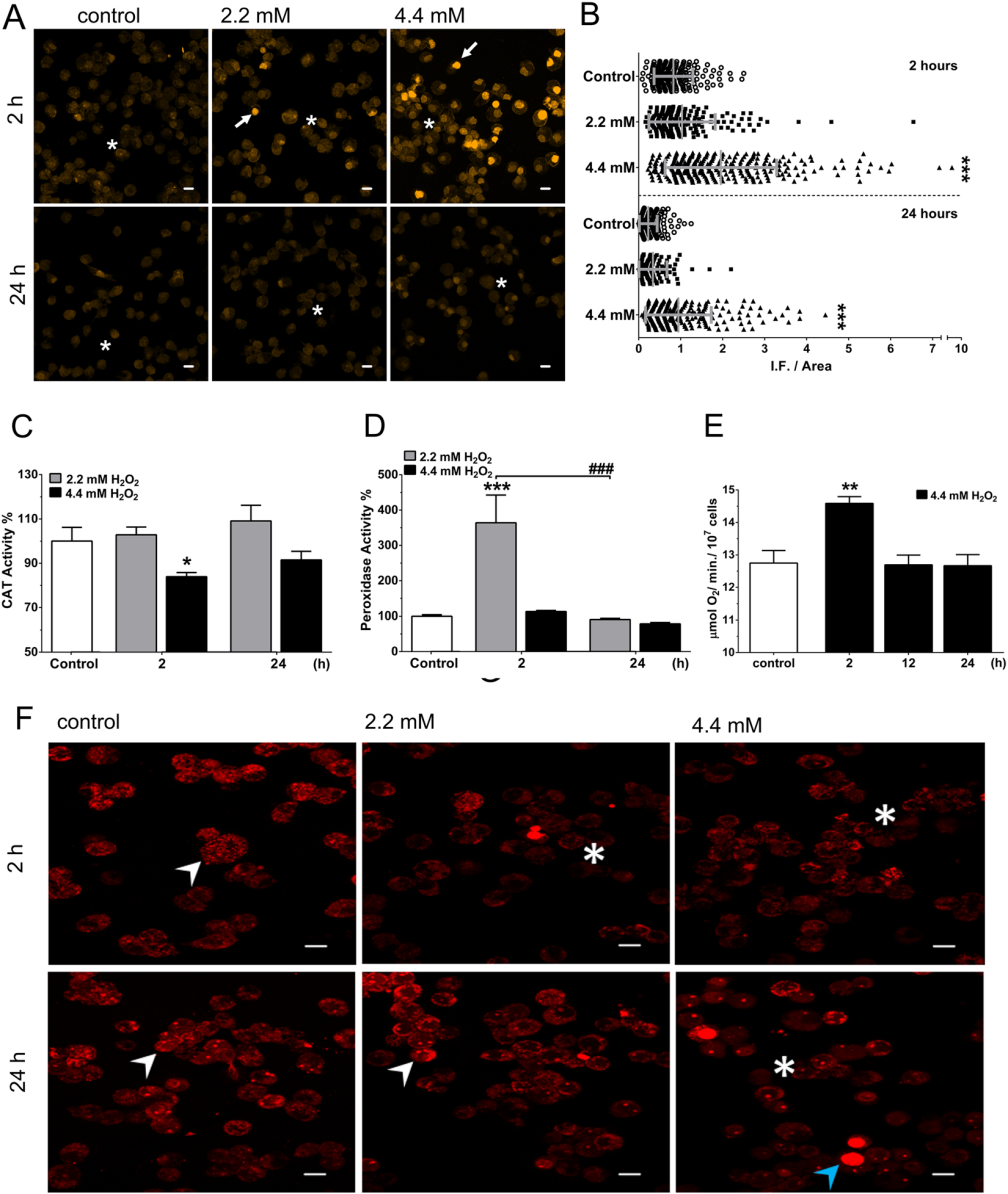
H_2_O_2_-tolerant BME26 cells show an adaptive response where ROS-scavenging induces ROS reduction over time, with a transitional increase in total O_2_ consumption. (**A**) ROS generation in short (2 h) and prolonged (24 h) H_2_O_2_ challenge at 2.2 mM and 4.4 mM was observed using DHE fluorescent probe and laser scanning confocal microscope LSM 710; scale bar: 10 µm. (**B**) DHE fluorescence intensity measured using Zen2011 Zeiss software. Presence of catalase (CAT) activity (**C**) and total peroxidase activity using AmplexRed peroxidase assay kit (Invitrogen) (**D**), the enzymatic activities were calculated in percentage, relative to control. (**E**) Rate of total oxygen consumption was measured in 10^7^ BME26 cells μmol/min using liquid-phase Oxytherm electrode (Hansatech) after 2, 12 and 24 h at 4.4 mM H_2_O_2_. Mitochondrial superoxide levels using MitoSox (**F**) were measured 2 h and 24 h after 2.2 mM or 4.4 mM H_2_O_2_ addition. The experiments were performed with three independent biological samples in three experimental replicates each, where ^∗^*p* < 0.05, ^∗∗^*p* < 0.01, ^∗∗∗^*p* < 0.001, compared to control; and ^###^*p* < 0.001 comparison over time, in Tukey’s multiple comparisons test.

Superoxide anions were quantified in H_2_O_2_-challenged BME26 cells using dihydroethidium (DHE) fluorescent probe and used as a measure of Reactive Oxygen Species (ROS) level^40^ (Fig. 4A). The mean fluorescence intensity (F.I.) per cell area in mm^2^, calculated by the software ZEN 2.3, is shown in Fig. 4B. Two hours after addition of 2.2 mM H_2_O_2_, ROS labeling was subtly greater than control, but did not show statistical difference in the quantitative analysis; ROS levels were equivalent to the untreated control condition after 24 h. However, following the treatment with 4.4 mM H_2_O_2_, a significantly increased ROS production was detected at 2 h, which decreases after 24 h of exposure. These findings suggest that the transient increase in ROS levels observed after the 2 h treatment was caused by the oxidative potential induced by exogenous H_2_O_2_ bolus addition. The elevated ROS levels were not sustained 24 h after H_2_O_2_ addition, and since BME26 cells remain viable under these conditions, it indicates that ROS scavenging is associated with protective mechanisms, suggesting that one or more ROS defense system could be involved (Fig. 4). Indeed, the relative transcription (RQ) of tick catalase, an enzymatic defense system, showed a significant increase 24 h after H_2_O_2_ challenge with either 2.2 mM or 4.4 mM (Supplementary Fig. S2A), but catalase activity was not significantly increased under any of the treatment conditions (Fig. 4C). On the other hand, according to AmplexRed detection kit, peroxidase activity was significantly increased after 2 h treatment at 2.2 mM H_2_O_2_, but not at LD_25_ (4.4 mM H_2_O_2_) (Fig. 4D). Because of an apparent discrepancy between the results regarding catalase expression and activity, we decided to assess the importance of the catalase system by challenging cells with H_2_O_2_ after catalase inhibition using classical inhibitor Aminotriazole (AT) (Supplementary Fig. S2B). After 4 h incubation with catalase inhibitor, BME26 cells were challenged for 24 h with 2.2 mM or 4.4 mM H_2_O_2._ We observed that cell viability was unaffected by the treatment at 2.2 mM, whereas at 4.4 mM there was a lethality of 90% (Supplementary Fig. S2B). Therefore, we observed that there is a great contribution of peroxidases in the non-lethal concentration (2.2 mM H_2_O_2_). On the other hand, at LD_25_ (4.4 mM H_2_O_2_), the results demonstrate that presence of catalase activity is essential for cell survival.

Another stress response enzyme previously described in ticks as an important factor for acaricide resistance in *R. microplus* is the phospholipid-hydroperoxide glutathione peroxidase (PHGPx)^41^. This enzyme is known for its role in cellular protection associated with reduction of hydroperoxides, thus preventing lipid peroxidation under conditions of oxidative stress. Increased PHGPx transcript levels were observed in response to 2.2 mM or 4.4 mM H_2_O_2_ after 24 h (Supplementary Fig. S2C), indicating a putative involvement of ROS control mechanisms by PHGPx in BME26 H_2_O_2_ tolerance.

Oxygen is the final electron acceptor in the mitochondrial electron transport chain during aerobic metabolism^42^. In BME26 cells, oxygen consumption significantly increased after treatment with 4.4 mM H_2_O_2_ (Fig. 4E), indicating a mechanism associated with metabolic cellular respiration. At a given density (10^7^ cells/mL), BME26 cells consumed oxygen at a rate of 12.5 µmol/min under control conditions; two hours after addition of 4.4 mM H_2_O_2_, oxygen consumption increased to 14.5 µmol/min. Interestingly, at 12 h and 24 h post-treatment, oxygen consumption returned to the same levels observed in control culture. The 2-h H_2_O_2_ treatment also promoted changes in mitochondrial superoxide production. Using a mitochondrial superoxide marker (MitoSox), a strong reduction of signal in H_2_O_2_ exposed cells (Fig. 4F, white asterisks) was observed when compared with the control condition (white arrowhead). Interestingly, after 24 h of exposure to 2.2 mM H_2_O_2_, mitochondrial superoxide levels were restored to the basal control condition, and at LD_25_ (4.4 mM H_2_O_2_) an intense increase in MitoSox signal was observed in a few cells (Fig. 4F, blue arrowhead). These results indicate that after 2 h of challenge, a tolerance mechanism combines ROS homeostasis with metabolic cellular respiration. In an oxidative extracellular environment situation caused by addition of exogenous H_2_O_2_, the increase in O_2_ consumption, with reduced mitochondrial superoxide levels, indicate that adaptation mechanisms lead to a greater flow in the electron transport chain, avoiding the escape of electrons and consequently the formation of mitochondrial superoxide.

### Glucose uptake and metabolism in H_2_O_2_-challenge BME26 cells

**Figure 5:**
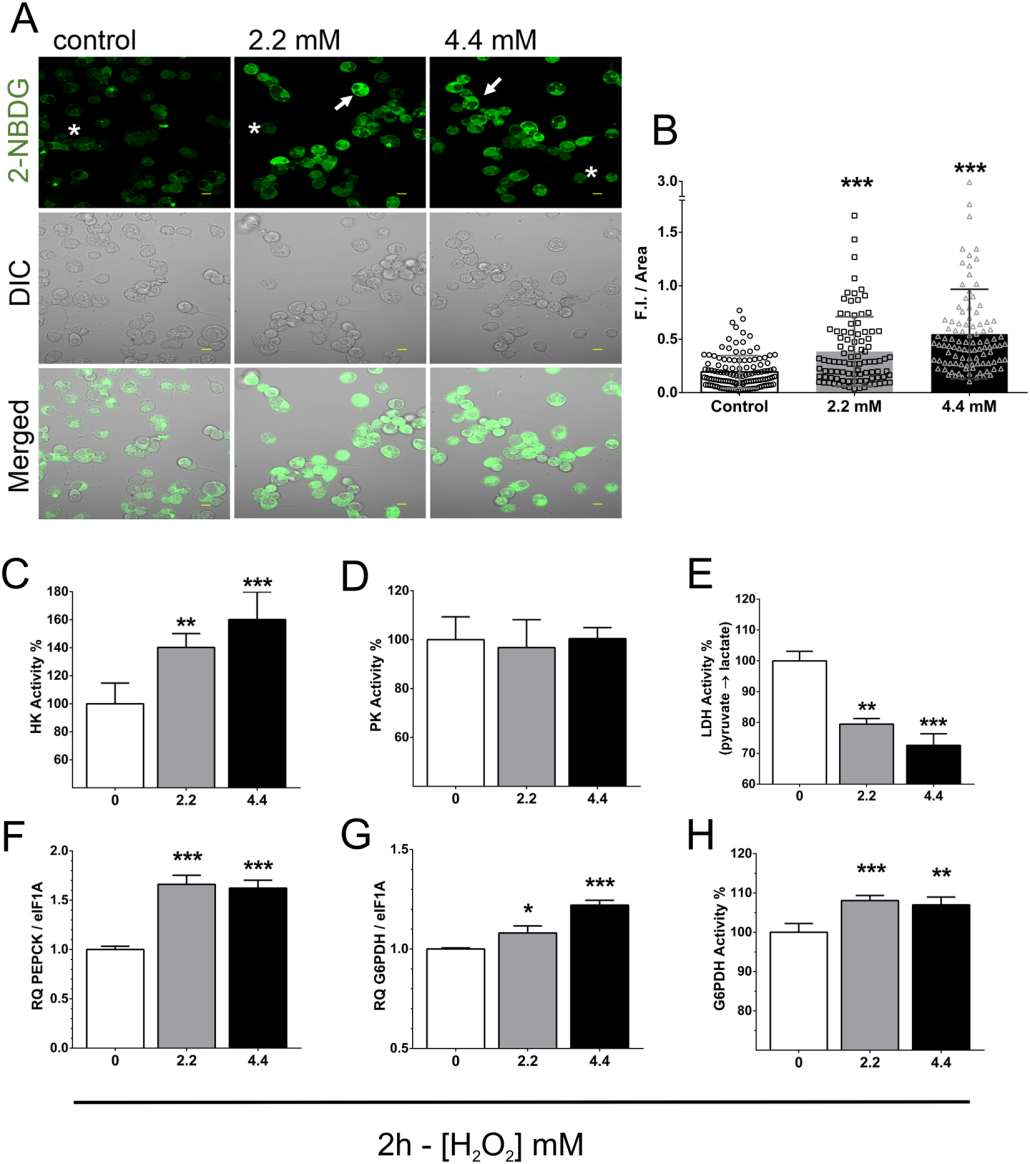
Glucose metabolism response induced by H_2_O_2_ in BME26 cells. (**A**) 2-NBDG glucose analog uptake after treatment with 2.2 mM or 4.4 mM H_2_O_2_ for 2 h, observed using laser scanning confocal microscope LSM 710. Scale bar: 10 µm. (**B**) Quantification of 2-NBDG glucose analog uptake by fluorescence intensity using Zen2011 Zeiss software. (**C**) Hexokinase (HK) activity, (**D**) pyruvate kinase (PK) activity, (**E**) lactate dehydrogenase (LDH) activity, (**F**) phosphoenolpyruvate carboxykinase (PEPCK) transcription, glucose 6-phosphate dehydrogenase (G6PDH) transcription (**G**) and activity (**H**) were measured 2 h after 2.2 mM or 4.4 mM H_2_O_2_ addition. Enzymatic activities were calculated in percentage, relative to control. Relative quantification (RQ) of metabolism genes was determined by real-time PCR. The experiments were performed with three independent biological samples in three experimental replicates each, where ^∗^*p* < 0.05, ^∗∗^*p* < 0.01, ^∗∗∗^*p* < 0.001, compared to control using Tukey’s multiple comparisons test.

Metabolic profiling of BME26 cells after H_2_O_2_ challenge indicates interesting alterations in carbohydrate metabolism towards sustaining the redox balance. The relationship between oxidative stress regulation and energy metabolism has been already suggested^43^. Also, components of the glycolytic pathway were previously shown to be modulated by H_2_O_2_^6^. Particularly, redox balance via NADPH production in metabolic pathways plays an important role in cell survival under oxidative conditions^6^. In the present work, glucose uptake was measured using its fluorescent analog 2-NBDG (2-[N-(7-nitrobenz-2-oxa-1,3-diazol-4-yl)amino]−2-deoxy-d-glucose), 2 h after H_2_O_2_ addition at 2.2 or 4.4 mM. Increased glucose uptake was detected as an intense fluorescent labeling was observed when compared with control cells (Fig. 5A). Quantitative analysis is presented as mean fluorescence intensity (F.I.) by area in mm^2^ (Fig. 5B). BME26 cells have been previously shown to be responsive to insulin signaling^10,44^, and we have successfully used insulin as a positive control in 2-NBDG internalization experiment (Supplementary Fig. S3).

Previous work using BME26 cell line identified an expressive increase in the transcription of genes involved in glycolysis and gluconeogenesis after challenge using high concentration of glucose^45^. Interestingly, hexokinase (HK) exhibited a higher activity 2 h after H_2_O_2_ challenge at both concentrations, 2.2 mM and 4.4 mM (Fig. 5C). In contrast, pyruvate kinase (PK) activity remained unaltered during the same treatment (Fig. 5D). We also observed minor changes at the transcript amounts for HK and PK. Relative transcription of HK remained unaltered 2 h after H_2_O_2_ challenge (Supplementary Fig. S4A). PK relative transcription was increased in BME26 cells after 2 h treatment with 2.2 mM H_2_O_2_ (Supplementary Fig. S4C), however, 24 h after H_2_O_2_ challenge, relative transcription of both HK and PK were reduced (Supplementary Fig. S4B,D). These data suggest that a differential relationship between transcriptional and enzymatic regulation of HK and PK is taking place under the tested conditions^45^.

**Figure 6:**
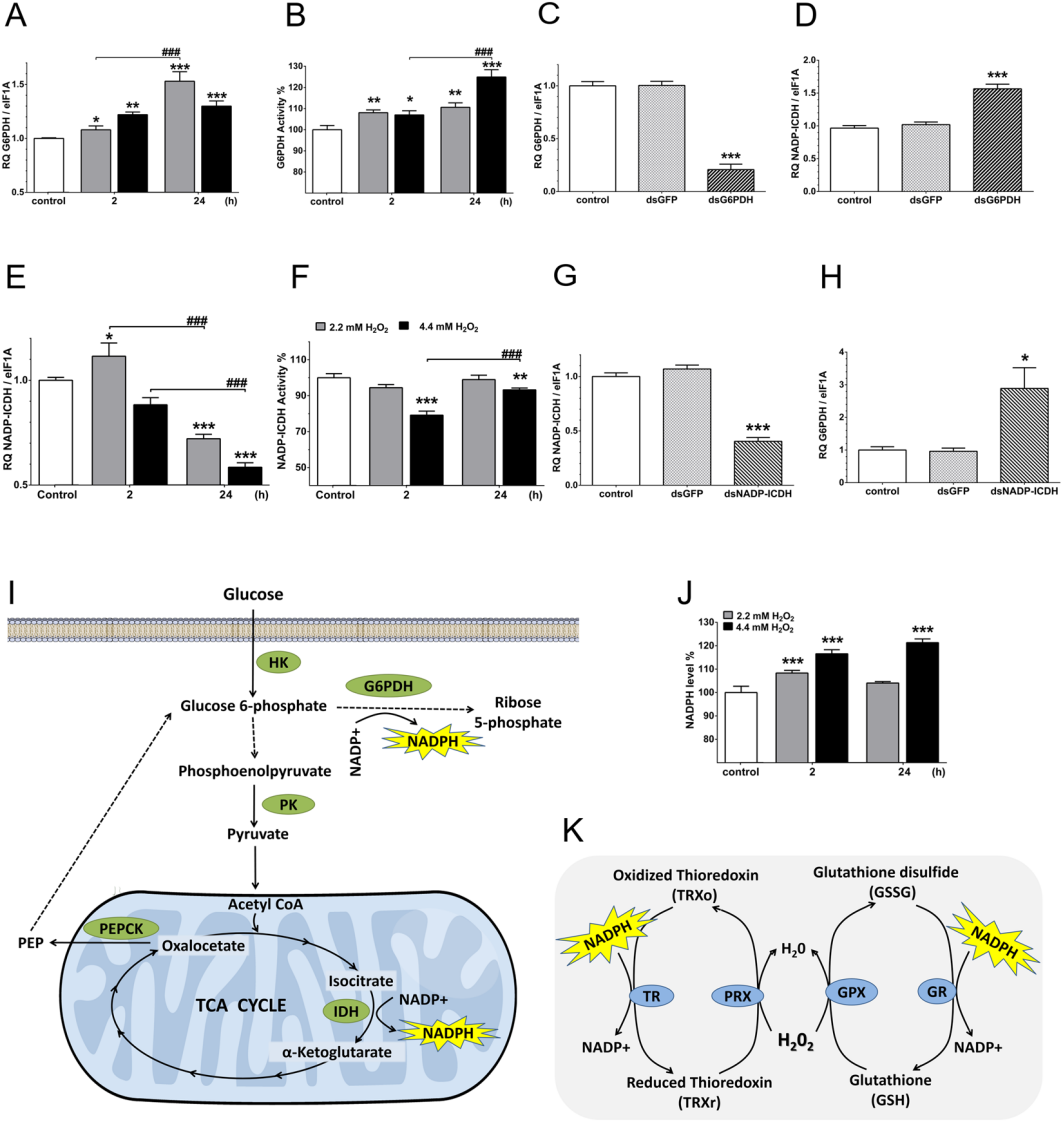
H_2_O_2_ tolerance derived from glucose metabolism and NADPH production in BME26 cells after H_2_O_2_ challenge. (**A**) Glucose 6-phosphate dehydrogenase (G6PDH) transcript level and (**B**) activity were measured 2 h and 24 h after 2.2 mM or 4.4 mM H_2_O_2_ addition. (**C**) Confirmation of G6PDH knockdown (78%) 3 days after incubation with 4 µg of *R. microplus* G6PDH dsRNA. (**D**) Relative quantification (RQ) of NADP-ICDH gene after G6PDH knockdown. (**E**) NADP^+^-dependent isocitrate dehydrogenase (NADP-ICDH) transcript amount and (**F**) activity were measured 2 h and 24 h after 2.2 mM or 4.4 mM H_2_O_2_ addition. (**G**) Confirmation of NADP-ICDH knockdown (60%) 3 days after incubation with 4 µg of NADP-ICDH dsRNA. (**H**) Relative quantification (RQ) of G6PDH gene after NADP-ICDH knockdown. (**I**) Metabolic adaptation scheme to maintain NADPH production, showing the pathways involving the presently analyzed enzymes related to NADPH production in BME26 cells after H_2_O_2_ challenge (Abbreviations: G6PDH, glucose 6-phosphate dehydrogenase; HK, hexokinase; NADP-ICDH, NADP^+^-dependent isocitrate dehydrogenase; PEP, phosphoenolpyruvate; PEPCK, phosphoenolpyruvate carboxykinase; PK, pyruvate kinase; TCA, tricarboxylic acid). (**J**) NADPH levels were measured 2 h and 24 h after 2.2 mM or 4.4 mM H_2_O_2_ addition. (**K**) Proposed mechanism of H_2_O_2_ scavenging using NADPH reducing potential (Abbreviations: GPX, glutathione peroxidase; GR, glutathione reductase; PRX, peroxiredoxin; TR, thioredoxin reductase). Experiments were performed with three independent biological samples in three experimental replicates each, where ^∗^*p* < 0.05, ^∗∗^*p* < 0.01, ^∗∗∗^*p* < 0.001, compared to control; and ^##^*p* < 0.01, ^###^*p* < 0.001 compared between the groups, in Tukey’s multiple comparisons test.

In some tissues under aerobic conditions or low oxygen (muscle hypoxia, for example), pyruvate is reduced to lactate by lactic fermentation^46^. In BME26 cells 2 h after challenged with H_2_O_2_, total O_2_ consumption increased; accordingly, lactate dehydrogenase activity was decreased under both H_2_O_2_ concentrations tested (Fig. 5E). This set of results indicates an aerobic oxidation fate for pyruvate. Phosphoenolpyruvate carboxykinase (PEPCK) was previously described as stimulated by increased transcription/translation of the gene (Hanson and Reshef, 2003; Yang *et al*., 2009). Interestingly, an increased PEPCK transcription was observed in both H_2_O_2_ treatment conditions (Fig. 5F), indicating a reloading of glucose 6-phosphate (G6P) through gluconeogenesis to maintain the activity of glucose-6-phosphate dehydrogenase (G6PDH). G6PDH relative transcript level and activity increased in BME26 cells 2 h after either H_2_O_2_ treatments in comparison with control conditions (Fig. 5G,H). This positive G6PDH response at both relative transcription and activity levels remained until 24 h after treatment with H_2_O_2_ (Fig. 6A,B). One of the cellular routes for G6P oxidation is the pentose phosphate pathway (PPP), a set of reactions that produce ribose 5-phosphate and reducing potential in the form of NADPH as major products, and is regulated by G6PDH.

### NADPH compensation involving G6PDH and NADP-ICDH to maintain redox balance in BME26 cells under oxidative environment

To investigate the importance of G6PDH for tolerance to H_2_O_2_ in BME26 cells, we generated a G6PDH knockdown (Table 1) in this cell line. A reduction of 78% in relative gene transcript level was observed (Fig. 6C). G6PDH activity was reduced in 42% 3 days after dsRNA addition, and this reduction was still seen 6 days after dsRNA-treatment (Supplementary Fig. S5A). G6PDH silencing did not affect BME26 cell viability in the absence of exogenous H_2_O_2_ (Supplementary Fig. S5B), nevertheless, it caused an increased NADP^+^-dependent isocitrate dehydrogenase (NADP-ICDH) relative transcription (Fig. 6D), which indicated a possible compensation mechanism to sustain NADPH production following G6PDH knockdown. NADP-ICDH is another NADPH-producing enzyme, which catalyzes the oxidative decarboxylation of isocitrate, producing alpha-ketoglutarate, CO_2_ and NADPH^25,26^. Its nucleotide sequence (accession number KY953209) was found in a *R. microplus* transcriptome database (RmINCT-EM) created by our research group using Illumina sequencing (BioProject ID PRJNA232001 at Transcriptome Shotgun Assembly (TSA) database – GenBank). The deduced amino acid sequence alignment showed a high conservation in critical residues (data not shown) and more than 88% identity to homologs in other ticks. Cells treated with G6PDH dsRNA for three days and submitted to H_2_O_2_ treatment at 2.2 mM (non-lethal concentration) or 4.4 mM (LD_25_ concentration) showed viability profiles similar to controls treated with unrelated-dsRNA (Fig. S5C). In addition, G6PDH chemical inhibition by 6-ANAM did not affect the viability of the cells following H_2_O_2_ challenge (Supplementary Fig. S5D), indicating that BME26 cells can use G6PDH-independent pathways for redox control in the presence of high concentrations of H_2_O_2_.

**Table 1:** GenBank accession numbers and oligonucleotide sequences used for RQ-PCR and dsRNA synthesis.

Gene	GenBank Access Number	Primers	Amplicon Size (bp)
*Glucose-6-phosphate Dehydrogenase* (G6PDH)	EU595878.1	5′-CGC AAC GAA TTG GTA TTG AGG-3′ 5′-CGA CTG CCA TAG GTG AGA TCC-3′	122
*NADP*^+^*-dependent isocitrate dehydrogenase* (NADP-ICDH)	KY953209	5′-CTT CAA AGC AGG TCT TAT GG-3′ 5′-AGG AAC GGG AAT ATC AAC TC-3′	128
*Catalase* (CAT)	KY953208	5′-GAG GAG AGG GAC CGC CTT AC-3′ 5′-GTG CCT TGG TGA AGT TCG TG-3′	97
*Hexokinase* (HK)	KF951259	5′-CAT GGA CAA AGA GCT TCA ACT GCT C-3′ 5′-GGA AAG CTC CCT TGA CCA GGG TA-3′	150
*Pyruvate kinase* (PK)	KF951260	5′-GGG CAA GAG GGC AAG ACA ACT G-3′ 5′-CAC GTT GAG CAC CTT GGT GAT G-3′	141
*Phospholipid-hydroperoxide Glutathione Peroxidase* (PH-GPx)	DQ180067	5′-GCG TCC TCC ATC TAT GAC TTC-3′ 5′-CTT GTT GGT CTT TCC TCA CTT G-3′	123
*Phophoenolpyruvate carboxykinase (PEPCK)*	KF951261	5′-CAAGCAATGAGTGCCTGCCAC-3′ 5′-ACAGTCTTCCGTTTTCATCTTG-3′	147
*Elongation factor 1-alpha* (ELF1A, Nijhof *et al*., 2009)	EW679365	5′-CGT CTA CAA GAT TGG TGG CAT T-3′ 5′-CTC AGT GGT CAG GTT GGC AG-3′	108
**Target gene**		**Primers**	
*dsG6PDH (553* *bp)*		5′- TAATACGACTCACTATAGGGGACCCGTGTTGTGATTGAG -3′ 5′- TAATACGACTCACTATAGGGCGCTCGTTGTTGATGTAGG -3′	
*dsNADP-ICDH (565* *bp)*		5′- TAATACGACTCACTATAGGGTGCGATCAAGAAGTACAACG -3′ 5′- TAATACGACTCACTATAGGGAAGGCGATGCTCATACCAC -3′	
*dsGFP (600* *bp)*		5′- TAATACGACTCACTATAGGGCCACGTCAAGCACCACCACC -3′ 5′- TAATACGACTCACTATAGGGGCGTAGTTTTCCTTATCGCG -3′	

*T7 promoter sequence (underlined).

**All primers are designed for Rhipicephalus microplus sequences unless otherwise specified.

Additional measurements of NADP-ICDH transcript levels and enzyme activity were performed on BME26 cells challenged with H_2_O_2_. NADP-ICDH relative transcription increased in BME26 cells 2 h after treatment with 2.2 mM H_2_O_2_, whereas a reduction of approximately 30% was observed after 24 h (Fig. 6E). However, NADP-ICDH enzyme activity in response to short or long exposure to H_2_O_2_ did not correlate to the changes in transcript levels. At 2.2 mM H_2_O_2_, the enzyme activity remained similar to the control, whereas at 4.4 mM a reduction of ~15% in NADP-ICDH activity was observed (Fig. 6F). We also performed gene silencing of NADP-ICDH (Table 1) in BME26 cells, with approximately 60% reduction in relative NADP-ICDH gene transcript level (Fig. 6G), which correlated with increased levels of G6PDH relative transcription (Fig. 6H). These observations suggest a compensatory mechanism involving NADPH-producing metabolic enzymes, which might contribute to maintain redox balance in BME26 cells under H_2_O_2_ treatment. A schematic representation summarizes the proposed cell adaptation to maintain NADPH production and H_2_O_2_ tolerance (Fig. 6I).

Increased NADPH content was observed in BME26 cells in response to H_2_O_2_ treatment for 2 h, and even after 24 h of treatment, the cells challenged with 4.4 mM still maintained a higher NADPH content than the control (Fig. 6J). It is possible that such an adaptive response is regulated by changes in gene expression of NADP^+^ reducing enzymes. Altogether, these results strongly suggest that glucose is taken up after H_2_O_2_ challenge, converted to glucose-6-phosphate and led mainly through the PPP. There is also a correlation between glucose uptake and increased NADPH content, which corroborates HK activity within PPP. Moreover, the present data suggest that enhanced NADPH production correlates with glucose metabolism in H_2_O_2_-challenged BME26 cells. Finally, the role of peroxidase activities in oxireduction reactions during the process of H_2_O_2_ tolerance is depicted in Fig. 6K, showing peroxiredoxin (PRx) and glutathione peroxidase (GPx) as examples. Maintaining the redox balance in this context requires a reduced substrate that can be oxidized by H_2_O_2_, as well as reductase enzymes (such as TR, thioredoxin reductase; and GR, glutathione reductase) to reduce the oxidized substrate. This reaction is only possible with the participation of a reducing agent such as NADPH, a product of the metabolic pathways analyzed in BME26 cells after H_2_O_2_ challenge.

## Discussion

In this work we demonstrate that BME26 cells have a metabolic response to oxidative challenge that leads to tolerance while maintaining redox balance. In aerobic organisms, ROS production is maintained under control by antioxidant systems^4,27,47,48^. ROS are involved in processes as diverse as signal transduction, pathogen killing, and gene regulation^49–52^. During oxidative stress, ROS cause cellular and tissue damage by reacting with biomolecules, which may trigger the development of diseases such as diabetes, sepsis, and neurodegenerative diseases^28,53,54^. However, oxidative compounds, including H_2_O_2_, are naturally formed in cells, and many researchers have suggested that ROS are also required for physiological redox signaling, which is essential to regulate certain cellular functions^55,56^. For instance, sustained ROS generation is required for successful tadpole regeneration in *Xenopus*, and to regulate Wnt/β-catenin signaling^57^. It has been postulated that adaptive responses to H_2_O_2_-induced stress in sensitive cells are primarily related to the induction of antioxidants enzymes, with emphasis on catalases, peroxidases and superoxide dismutases^27^. Induction of these enzymes is essential for ROS scavenging and is partly responsible for promoting tolerance towards H_2_O_2_.

Previous studies demonstrated that *D. melanogaster* fed with hydrogen peroxide at 0.1% (29.4 mM) had a significant mortality^31^. *In vitro*, when arthropod and mammalian cells were exposed to increasing H_2_O_2_ concentrations (Fig. 2), the arthropod embryonic cell lines (BME26, Aag2 and S2) displayed higher H_2_O_2_ tolerance compared to mammalian cells (Fig. 2A–D and S1 A,B). Interestingly, H_2_O_2_ tolerance by S2 *Drosophila* cells (Fig. S1B) is in agreement with previous publications showing an increase in expression of oxidative stress resistance genes and NADPH levels in *Drosophila* under diverse stress conditions, such as starvation, paraquat treatment, hyperoxia, hypoxia, and desiccation^25,58,59^. BME26 cells had an LD_50_ near 6 mM H_2_O_2_ after 24 h of exposure, whereas AH927 cells, as well as other mammalian cells investigated in this study, have LD_50_ of 0.2 mM H_2_O_2_. H_2_O_2_ was previously reported to stimulate Akt phosphorylation and promote mammalian cell survival at 20 to 50 µM concentrations^3^. We observed that BME26 cells tolerated and retained their proliferative capacity even after treatment with 4.4 mM H_2_O_2_ (Fig. 3). These findings indicate a remarkable H_2_O_2_ tolerance by ticks, which could be a result of regulated mechanisms comprising ROS generation, scavenging, and signaling^60,61^.

Intracellular ROS content in BME26 cells after 2 h of H_2_O_2_ treatment has shown to be increased in a dose-dependent manner. However, 24 h later, ROS were no longer detected, suggesting a possible scavenging mechanism (Fig. 4A,B). Catalase, which decomposes H_2_O_2_ into H_2_O and oxygen, is in the first line of defense in cells undergoing oxidative stress, and it was previously described as a highly active component during increased oxygen consumption in both *R. microplus* eggs and larvae^62^. According to the results presented here, although catalase transcription increased following H_2_O_2_ challenge, catalase activity was unchanged 24 h after H_2_O_2_ bolus addition (Figs 4C and S2A). However, when catalase was inhibited by AT, BME26 cells showed to be very sensitive to 4.4 mM H_2_O_2_ concentration (Fig. S2B). BME26 cells with inhibited catalase were not sensitive to 2.2 mM H_2_O_2_, which corroborates the hypothesis that increased peroxidase activity (Fig. 4D) maintains tolerance. Taken together, these data reveal that catalase activity participation as a response to oxidative conditions is not as evident in tick cells as it is observed in mammals^63^, which suggests that other enzymes could be taking part in this adaptive process. Another hypothesis is that basal peroxidase and catalase enzymatic activities present in BME26 cells are sufficient for a rapid response to exogenous H_2_O_2_. To address this, a detailed study of enzyme kinetics for catalases and peroxidases in BME26 cells is needed, as well as the assessment of these activities in periods shorter than 2 h.

After 2 h of H_2_O_2_ addition we observed an increase in O_2_ consumption (Fig. 4E), whereas the mitochondrial superoxide content diminished (Figs 4F). In addition, a conserved mechanism coupling ROS homeostasis to metabolic cellular respiration seems to take place after 2 h incubation with H_2_O_2_. This oxidative response is probably associated with mitochondrial membrane potential (∆Ψm) for ROS production, and inversely correlated to ATP synthesis and ADP depletion^8,9^. In this work, the relationship between oxygen consumption (Fig. 4E) and glucose uptake (Fig. 5A,B), with simultaneous decrease in mitochondrial superoxide activity (Fig. 4F), is presented as an important adaptive cellular strategy in response to oxidative environment. In this context, hexokinase and G6PDH were shown to be the main enzymes participating in the first two hours of this cellular adaptation (Fig. 5C,H). Our data show that NADPH formation (Fig. 6J) might represent an anti-oxidative defense, shifting glucose to PPP for oxidant detoxification, mainly by the action of G6PDH, a scenario that does not exclude the participation of other enzymes in the anti-oxidative response in glycolytic cells.

The recruitment of HK to the mitochondria diminishes ROS production by increasing the efficiency of electron transfer during aerobic respiration^8,9^. HK is known to be released from the surface of mitochondria by its enzymatic product, G6P^6,8,9,22,43,58^. This system can control mitochondrial ROS emission in glycolytic cells by providing NADPH and maintaining HK activity (Fig. 5), meaning glucose regulates enzymatic sources of intracellular NADPH in BME26 cells. Indeed, results by da Silva *et al*.^4^ indicated that mitochondrial HK activity performed a key preventive role against oxidative stress, reducing mitochondrial ROS generation through an ADP-recycling mechanism.

Another enzyme investigated in this study was the phosphoenolpyruvate carboxykinase (PEPCK), which occupies a key position in energy homeostasis because it is involved in the regulation of fatty acid re-esterification, glucose synthesis, transamination and the cataplerosis of citric acid cycle anions^64^. It is well established that alterations in PEPCK gene transcription regulate the total activity of this enzyme^64,65^. Relative transcription of PEPCK increases 24 h after H_2_O_2_-challenge (Fig. 5F), suggesting an increased activity at this time-point. Pyruvate produced by glycolysis can be used to generate G6P through gluconeogenesis, a process regulated by PEPCK^66,67^. The increased G6PDH activity 2 h and 24 h after H_2_O_2_ challenge (Fig. 6B) demands higher concentration of glucose-6-phosphate (G6P) to ensure the NADPH production necessary to cope with oxidative stress (Fig. 5). Therefore, in this condition, both glycolysis and part of gluconeogenesis are activated at same time.

In G6PDH knockdown BME26 cells, a cellular environment was induced by oxidative stress in which NADP-ICDH seems to be a compensatory NADPH provider (Fig. 6). Since G6PDH transcription and activity increased after H_2_O_2_ challenge (Fig. 6A,B), a high energy load is necessary to supply sufficient G6P for pentose phosphate pathway, without affecting cellular energy metabolism. Thus, the activity of NADP-ICDH (Fig. 6F) and NADP-ME (malic enzyme, Data not shown) are expected to be at the level of the control cells, indicating a less costly adaptation mechanism. Studies in other organisms have shown that the expression of NADP-ME is regulated by stress factors. For example, in *Drosophila*, the interaction of NADPH-producing enzymes (G6PDH, NADP-ICDH and NADP-ME) under different stress conditions, such as oxidative stress, starvation, and desiccation, was evaluated. The results showed that NADPH production was mostly afforded by G6PDH and NADP-ICDH, which were more accentuated in oxidative stress, and by NADP-ME under starvation stress^25^. Thus, we formulate two hypotheses for a smaller participation of NADP-ME in BME26 cells: I) the enzyme is mainly required in a situation of low energy load, or II) there is a modest contribution of this enzyme to the oxidative balance when compared to G6PDH, for example, maintaining the oxidative balance in mitochondrial metabolism. Mitochondrial ROS homeostasis is supported by different pathways; those have been suggested as potential targets to eliminate cancer cells through increased ROS formation under NADPH deficient conditions^68^. Either NADH or NADPH are required as co-factors for anti-oxidative enzymatic activities^24^. An additional adaptive mechanism displayed by BME26 in response to H_2_O_2_ was the upregulation of NADP^+^ reducing enzymes, which increases intracellular NADPH (Fig. 6I). According to these results, BME26 cells treated with H_2_O_2_ induce NADPH production as an adaptive strategy of compensation between NADP-ICDH and G6PDH enzymes^26,69^.

A great progress has been achieved with arthropod model systems to study various human disorders including diabetes, multiple sclerosis, and epilepsy^70–72^. Thus, arthropod cell lines act as excellent experimental models to study physiology, gene regulatory networks, metabolic fluxes, and the regulation of energy homeostasis^11–14,37,44,73^. The present work demonstrates that ticks are able to support high H_2_O_2_ concentrations *in vivo* and represents the first analysis of an adaptive response to H_2_O_2_, where tolerance is linked to metabolic control in eukaryotic cells. Moreover, this study helps elucidate an adaptive mechanism developed by BME26 cells under H_2_O_2_ exposure to maintain cellular performance and redox balance. Taken together, the results contribute to a better understanding of tick physiology and metabolism. Finally, it is important to determine if the adaptive mechanism described here is a species-specific phenomenon or could also be extended to the physiology of other hematophagous arthropods.

## Methods

### Ticks and tick H_2_O_2_ treatment

*Rhipicephalus microplus* (Porto Alegre strain), were reared on Hereford calves (*Bos taurus taurus*) obtained from a naturally tick-free area (Santa Vitória do Palmar, RS, Brazil; 33°32′2″ S, 53°20′59″ W) maintained in individual sheds at the Faculdade de Veterinária of Universidade Federal do Rio Grande do Sul (UFRGS). During the off-host period of the life cycle, ticks were kept at 27 °C and 85% relative humidity. The experiments were approved and conducted following the guidelines of the Ethics Committee on Animal Experimentation of the same university.

To access tick susceptibility to H_2_O_2_, partially engorged adult females were manually removed from experimentally infested cattle. Ticks weighing between 25 mg and 65 mg were immobilized on a tray covered with double-sided adhesive tape and injected with 1 µL of H_2_O_2_ at 0.5, 1, 2, 3, 4 or 5 M concentrations (equivalent to the quantities of 0.5, 1, 2, 3, 4 and 5 µmol), using precision syringe (Hamilton). Groups of 33 females were assigned to each condition of 0, 0.5, 1 and 2 µmol; and 17 females for each condition of 3, 4 and 5 µmol. These ticks were artificially fed using microhematocrit capillary tubes filled with blood from non-infested bovines, collected in the presence of sodium citrate. Females were allowed to feed for approximately 24 h, and then kept at 27–28 °C and 80–90% relative humidity^74^. After this period, survival and reproduction were evaluated by measuring weight gain to determine blood feeding capacity, reproductive efficiency by determining the oviposition capacity, and eclosion rate.

### BME26 cell line and H_2_O_2_ treatment

BME26 tick embryo cell line was originally obtained as previously described^37^, and has been maintained in Leibovitz L-15 medium (Sigma-Aldrich®), supplemented with amino acids, glucose, mineral salts, and vitamins, according to^75^. The medium was diluted in sterile water (3:1), followed by addition of 10% tryptose phosphate broth (Sigma-Aldrich, #T8782), 10% fetal calf serum (Nutricell®, inactivated by heating), and commercial antibiotic Penicillin-Streptomycin (Gibco, #15140122) was diluted in the medium (1:100), according to the manufacturer’s instructions. To perform the experiments, cell culture density was standardized at an initial aliquot of 10^7^ cells per bottle (25 cm^2^) in 5 mL medium (2 × 10^6^ cells/mL), to be used 14 days later at an expected concentration of approximately 2 × 10^7^ cells per bottle (4 × 10^6^ cells/mL)^10^. Then, resuspended cells were counted and typically plated at 5 × 10^5^ cells in 500 µL of medium per well in a 24-well plate. Small modifications for indicated experiments were as follows: ^(1)^ for microscopy with fixed cells, 2.5 × 10^5^ cells in 500 µL of medium per well were seeded on round coverslips in a 24-well plate; ^(2)^ for microscopy with *in vivo* staining of intact cells (MitoSox and 2- NBDG), 2.5 × 10^5^ cells in 2 mL of medium were seeded in cell culture dish for confocal microscopy (SPL life sciences, Model: 20035); ^(3)^ for oxygen consumption test, the treatment was made in standardized bottle with approximately 2 × 10^7^ cells; ^(4)^ for assessing long-term viability (5 days), cells were seeded at 2.5 × 10^5^ cells in 500 µL of medium per well in a 24-well plate.

Treatment was performed with a single H_2_O_2_-bolus addition. To access cell susceptibility, cells were incubated with H_2_O_2_ at concentrations ranging from 62 µM to 1000 µM, and cell viability was checked 24 h after treatment. To determine LD_50_, cells were incubated with H_2_O_2_ at concentrations ranging from 2.2 mM to 13 mM, and cell viability was checked 24 h after treatment. The concentrations of 2.2 mM and 4.4 mM H_2_O_2_ were chosen for further experiments, with incubations in indicated periods (usually 2 h and 24 h). BME26 cells were used between passages 40–60.

### Cell lines used for comparative tests of H_2_O_2_ susceptibility

Aag2 cell line (continuous culture of *Aedes aegypti* embryo cells) and Schneider-2 (S2) cells (continuous culture of *Drosophila melanogaster* embryonic cell) were maintained in Schneider 2 medium with L-glutamine (Gibco, #21720024) supplemented with 10% fetal bovine serum (Nutricell®, inactivated by heating) and Penicillin/Streptomycin at 100 U/mL and 100 μg/mL, respectively (Gibco, #15140122). Cells were maintained at 28 °C, with culture medium replaced 2–3 times a week. For the experiment, cells were seeded in 5 × 10^5^ cells in 500 µL of medium per well in a 24-well plate.

Mammalian cells were provided by the cell culture division of the Carlos Chagas Filho Institute of Biophysics – UFRJ, only for performing a comparative H_2_O_2_ susceptibility test. Rhesus monkey kidney epithelial cells (LLC-MK2 continuous lineage) were maintained in Dulbecco’s modified Eaggle’s medium (DMEM) supplemented with 10% fetal bovine serum. For the experiment, the trypsinized and resuspended cells were counted and seeded at 4 × 10^5^ cells in 500 µL of medium per well in a 24-well plate. Peritoneal macrophages were obtained by intraperitoneal lavage of the C57Black/6 mouse. For the experiment, resuspended cells were counted and seeded at 8 × 10^4^ cells in 500 µL of medium per well in a 24-well plate.

To assess cell susceptibility, the treatment was performed with a single H_2_O_2_-bolus addition, at indicated concentrations ranging from 62 μM to 1000 μM and cell viability was verified 24 h after treatment.

### Cell Viability Assays

Cell viability was determined using a Neubauer hemocytometer with trypan blue exclusion technique and visual detection^76^. After H_2_O_2_ treatment, cells in each well were thoroughly rinsed with PBS and detached with 1 mL of a solution containing trypsin and trypan blue, in a proportion of 3.5:1:0.5 (v:v:v) of PBS, trypsin (2.5%, no phenol red, #15090046), and trypan blue 0.4% (Sigma-Aldrich). Ten microliters of the cell suspension were loaded into each chamber of the hemocytometer. The experimental procedure and calculation were done according to standard methodology^77^, focus on the grid corner regions of 1 × 1 mm dimension using a 10x objective to take images using camera Axiocam 503 color coupled to microscope Axio Imager 2 (Zeiss). Cells were counted using cell counter Manual Counting plugin on ImageJ software^78^.

Alternatively, cell viability was measured using the tetrazolium dye 3-(4,5-dimethylthiazol-2-yl)−2,5-diphenyltetrazolium bromide (MTT) assay to measure reduction to its insoluble formazan. Briefly, after H_2_O_2_ treatment, medium was replaced by 0.5 mL of fresh medium, and 50 µL of MTT (5 mg/mL in PBS) were added to each well. After 2 h of incubation at 34 °C, the medium was completely removed, and 1 mL of acid-isopropyl alcohol (0.15% HCl in isopropyl alcohol) was added to dissolve formazan crystals. The mixture was transferred to 1.5-mL tubes and centrifuged at 6000 × *g* for 15 min. The clear supernatant was collected for absorbance measurement at 570 nm in a UVmini-1240 UV-Vis spectrophotometer Shimadzu (Kyoto, Japan). Unless otherwise stated, the absorbance values of the control treatment were used for normalization (100% viability)^10^.

### Enzymatic Activities

#### Sample preparation

After H_2_O_2_ treatment, cells in each well of a 24-well plate were washed twice with 500 μl of PBS pH 7.0 and detached in PBS. Resuspended cells were transferred to an identified eppendorf tube and centrifuged at 6,000 rpm for 15 minutes at 15 °C (hettich-universal centrifuge 320). Three eppendorf tubes with the same treatment were pooled and lysed in 200 μl of the lysis buffer prepared with 980 μL of the Reaction Buffer (specific for each activity as described below); 10 μl of Triton-X100 (at the final concentration of 0.1%); 10 μL of protease inhibitor cocktail (Sigma aldrich #P8340). The homogenate was mixed by vortexing for 10 seconds, followed by mechanical lysis passing 20 times by the 1-ml syringe with 26 G needle. The cell lysate was centrifuged at 6,000 rpm for 10 minutes at 4 °C (hettich-universal centrifuge 320) to remove debris and intact cells, and the homogenate cell lysate supernatant was collected in a clean eppendorf tube, always kept on ice before assays.

#### Catalase (CAT) activity

Cell lysate (50 μL) was assayed for catalase (CAT) activity in 420 μL of 100 mM Tris-HCl Buffer, pH 8.0 and 230 μL of 60 mM H_2_O_2_. CAT activity was determined spectrophotometrically (UVmini-1240 UV-Vis Shimadzu - Kyoto, Japan) by monitoring the absorbance at 240 nm, at 25 °C. The reaction rate was determined by the consumption of H_2_O_2_ per minute, where one unit of enzyme is the amount needed to convert 1 mol of H_2_O_2_ in H_2_O + O_2_.

#### Glucose-6-phosphate Dehydrogenase (G6PDH) Activity

G6PDH activity was measured in 55 mM Tris-HCl Buffer containing 3.3 mM Mgl_2_ pH 7.8, 3.5 mM glucose 6-phosphate and 2 mM β-NADP^+^, in 700 µl final volume. The reaction was started with addition of 30 µl of homogenate cell lysate. The formation of β-NADPH was monitored spectrophotometrically at 340 nm, 30 °C (UVmini-1240 UV-Vis Shimadzu - Kyoto, Japan) during 7 minutes, using a molar extinction coefficient of 6.22 M^−1^.

#### NADP^+^-dependent isocitrate dehydrogenase (NADP-ICDH) Activity

NADP-ICDH activity was measured in 50 mM MOPS, containing 4 mM MgCl_2_ pH 8.0; 3.5 mM isocitrate and 2 mM β-NADP^+^, in 700 µl final volume. The reaction was started with addition of 30 µl of homogenate cell lysate. The formation of β-NADPH was monitored spectrophotometrically at 340 nm, 30 °C (UVmini-1240 UV-Vis Shimadzu - Kyoto, Japan) during 7 minutes, using a molar extinction coefficient of 6.22 M^−1^.

#### Hexokinase (HK) Activity

HK activity was assayed with 30 µL of homogenate cell lysate in 20 mM Tris–HCl pH 7.5 containing 6 m MgCl_2_, 1 mM ATP, 0.5 mM NAD + and 10 mM NaF. The enzymatic reaction was started with 2 mM glucose. The glucose 6-phosphate produced was measured indirectly by adding an equal volume of 1 unit /mL glucose 6-phosphate dehydrogenase from *Leuconostoc mesenteroides*, and 0.3 mM β-NAD^+^, in 700 µl final volume. The production of β-NADH was determined at 340 nm, 30 °C (UVmini-1240 UV-Vis Shimadzu - Kyoto, Japan) using a molar extinction coefficient of 6.22 M^−1^, as previously described^45^.

#### Pyruvate Kinase (PK) Activity

PK activity was measured using 30 µl of homogenate in 20 mM Tris–HCl pH 7.5, 5 mM MgCl_2_, 1 mM ADP, 0.4 mM NADH and 1 unit /mL lactate dehydrogenase, and the reaction was started with 1 mM phosphoenolpyruvate, in 700 µl final volume. The β-NADH consumption was evaluated spectrophotometrically (UVmini-1240 UV-Vis Shimadzu - Kyoto, Japan) at 340 nm, 25 °C using a molar extinction coefficient of 6.22 M^−1^, as previously described^45^.

#### Peroxidases Activity (Amplex-Red)

After treatment, 2 × 10^6^ cells were washed and suspended in 500 µL of PBS. Cell suspension was centrifuged at 6,000 rpm for 10 minutes (hettich-universal centrifuge 320). After the supernatant was discarded and the pelleted cells were suspended in 250 µL of lysis buffer (100 µL of 20 mM aminotriazole (Sigma-aldrich #A8056), 10 μL of protease inhibitor cocktail (Sigma-aldrich #P8340) in reaction buffer of Amplex Red kit (Invitrogen #A22188)). Lysis was mechanically induced by 20 cycles of aspiration and dispensing using a 1-ml syringe with 26 G needle. After centrifuging at 6,000 rpm for 5 minutes at 4 °C, the supernatant was collected for assay, which was performed according to the Peroxidase Assay manufacturer’s protocol. A standard curve was prepared with horseradish peroxidase (HRP) concentrations of 0 to 6 mU/mL, in 50 µL. Samples from each treatment were prepared in triplicate, with 50 µL of each homogenate being diluted in the provided kit’s reaction buffer. Reactions containing 50 µM Amplex Red reagent, 1 mM H_2_O_2_ and HRP or the samples in reaction buffer were incubated for 30 minutes at room temperature. Absorbance at 560 nm was then measured using Thermo Scientific Multiskan GO UV/Vis microplate reader. Background, determined from a control reaction in the absence of enzyme, was subtracted from each value.

#### Protein Determination and Calculation of Enzyme Activity

For calculation of specific enzymatic activities, total potein in samples was determined using Bicinchoninic Acid Protein Assay Kit (Sigma Aldrich, #BCA1), with Bovine Serum Albumin as standard. Enzymatic activity was normalized based on total protein quantification (U/mg total protein). After normalization, activity was expressed as percentage by setting the specific activity of each respective control condition as 100%.

### Statistical Analyses

Experiments were performed with three independent biological samples in three experimental replicates each, to obtain the mean. All values are expressed as mean ± S.D. Data were verified for normal distribution using Shapiro–Wilk test. When normality was confirmed, statistical significance was assessed by one-way and two-way ANOVA to determine significant differences between groups. The Tukey test was used to compare data between three groups. Significance was set at *p < 0.05, **p < 0.01, ***p < 0.0001. n.s., not significant (compared to control); and ^#^p < 0.05, ^##^p < 0.01, ^###^p < 0.0001 (compared within the same concentration between the different times 2 and 24 hours).

Image analysis was made based on three independent biological samples in three experimental replicates (coverslips) each. Three or more images from each replicate were recorded by laser scanning confocal microscope LSM 710, Zeiss. For further image analyses, the mean fluorescence intensity (F.I.) by cellular area in mm^2^ was calculated using the software ZEN 2.3 (blue edition) from Carl Zeiss Microscopy GmbH, 2011, or cell and nuclei counting was performed using Cell Counter plugin on ImageJ software^78^.

Percentage calculations, as well as statistical and graphical analyses were performed using GraphPad Prism 6 Software.

## Supplementary information

Supplementary video 1

Suplementary figures 1
