# Supplementary material for: Carbohydrate Metabolic Compensation Coupled to High Tolerance to Oxidative Stress in Ticks
Source: Sci Rep. 2019 Mar 18;9:4753. doi: 10.1038/s41598-019-41036-0 (PMC6427048; doi:10.1038/s41598-019-41036-0)
Supplement: Supplementary file 2 — Suplementary figures 1 [file 41598_2019_41036_MOESM2_ESM.pdf]

Carbohydrate Metabolic Compensation Coupled to High Tolerance to Oxidative Stress in Ticks

Bárbara Della Noce [a,e], Marcelle Vianna de Carvalho Uhl [a,e], Josias Machado [a,e], Camila Fernanda Waltero [a,e], Leonardo Araujo de Abreu [a,e], Renato Martins da Silva [c,e], Rodrigo Nunes da Fonseca [a,e], Cintia Monteiro de Barros [a], Gabriela Sabadin [b], Satoru Konnai [c], Itabajara da Silva Vaz Jr. [b], Kazuhiko Ohashi [c] and Carlos Logullo [a,d,e#]

[a] Laboratório Integrado de Bioquímica Hatisaburo Masuda and Laboratório Integrado de Morfologia, NUPEM-UFRJ, Macaé, RJ, Brazil.

[b] Centro de Biotecnologia and Faculdade de Veterinária – UFRGS, Porto Alegre, RS, Brazil.

[c] Laboratory of Infectious Diseases, Hokkaido University, Sapporo 060-0818, Japan.

[d] Instituto de Bioquímica Médica Leopoldo de Meis, Universidade Federal do Rio de Janeiro, Rio de Janeiro, RJ, Brazil.

[e] Instituto Nacional de Ciência e Tecnologia em Entomologia Molecular, Rio de Janeiro, RJ, Brazil

[#] Corresponding author, Carlos Logullo: carlos.logullo@bioqmed.ufrj.br.

SUPPLEMENTARY FIGURE LEGENDS

Supplementary Figure S1. Additional evidence of the tolerance of BME26 cells and *Drosophila* Schneider 2 (S2) cells to H_2O_2 challenge. (A) BME26 and (B) *Drosophila* Schneider 2 (S2) cell viability measured by MTT assay 24 hours after H_2O_2 treatment (Related to Fig. 2A-D). (C) BME26 cell viability measured by cell counting in hemocytometer with Trypan blue exclusion (Related to Fig. 2F). (D) BME26 morphology assessed by cytological analysis using Panoptic staining. Microscopy images were captured in bright field using Axio Scope. A1 Polarized Light Microscope, Zeiss and Zeiss software (Blue edition). Scale bar: 10 µm. Yellow arrows show the reduced-size cytosol 24 h after H_2O_2 treatment (Related to Fig. 2G). The experiments were performed with three independent biological samples in three experimental replicates each, $*p < 0.05$, $**p < 0.01$, $***p < 0.001$, compared to control in Tukey's multiple comparisons test.

Supplementary Figure S2: Additional H_2O_2-scavenging enzymes contribute to adaptive response. (A) Transcript levels of catalase (CAT) after 2.2 mM and 4.4 mM H_2O_2 treatment during 2 h and 24 h. (B) Catalase was inhibited by 3-amino-1,2,4-triazole (Aminotriazole, AT) for 24 h. (C) Phospholipid-hydroperoxide Glutathione Peroxidase transcript levels 2 h and 24 h after H_2O_2 treatment. Relative quantification (RQ) of gene transcription was determined by real-time PCR. The experiments were performed with three independent biological samples in three experimental replicates each, $*p < 0.05$, $***p < 0.001$, compared to control; and $\#\#p < 0.01$, $\#\#\#p < 0.001$ comparison over time, in Tukey's multiple comparisons test.

Supplementary Figure S3. Glucose uptake assay control using insulin. Glucose analog 2-NBDG uptake by cells in response to 1 mM insulin for 1 hour.

Supplementary Figure S4. Hexokinase and pyruvate kinase relative transcription in H_2O_2 -treated BME26 cells. Transcript levels of HK 2 h (A) and 24 h (B) after addition of 2.2 mM or 4.4 mM H_2O_2; and transcript levels of PK 2 h (C) and 24 h (D) after the same treatment. *R. microplus* elongation factor-alpha gene (Elf1A)[1] was used as reference for relative quantification (RQ). The experiments were performed with three independent biological samples in three experimental replicates each, $*p < 0.05$, $**p < 0.01$, $***p < 0.001$, compared to control; and $\#\#p < 0.01$, $\#\#\#p < 0.001$ comparison over time, in Tukey's multiple comparisons test.

Supplementary Figure S5. G6PDH chemical inhibition or knockdown did not alter H_2O_2-tolerance. (A) G6PDH activity was measured in BME26 cells 3 and 6 days after G6PDH knockdown. (B) G6PDH gene silencing did not affect BME26 cell viability in the absence of exogenous H_2O_2. (C) BME26 cells viability assessed by cell counting with trypan blue dye exclusion after G6PDH knockdown (3 days incubation with dsRNA), followed by challenge with 2.2 and 4.4 mM H_2O_2 for 24 h. The experiments were performed with three independent biological samples in three experimental replicates each, where $*p < 0.05$, $***p < 0.001$, compared to control; and $\#\#p < 0.01$, compared between the groups, in Tukey's multiple comparisons test. (D) BME26 cells were incubated with 6-ANAM (6-aminonicotinamide), a known G6PDH inhibitor, at concentrations of 100, 200 and 400 µM. After 10 min, cells were treated with H_2O_2 at 4.4 mM and 6.6 mM. This experiment was performed with three

independent biological samples in three experimental replicates each, ** $p < 0.01$, *** $p < 0.0001$, compared to the control without H_2O_2 (black bar) within each group of the same concentration of inhibitor; and # $p < 0.05$; ## $p < 0.01$; ### $p < 0.0001$ compared to the control without inhibitor within each group of the same H_2O_2 concentration, in Tukey's multiple comparisons test.

Figure S1

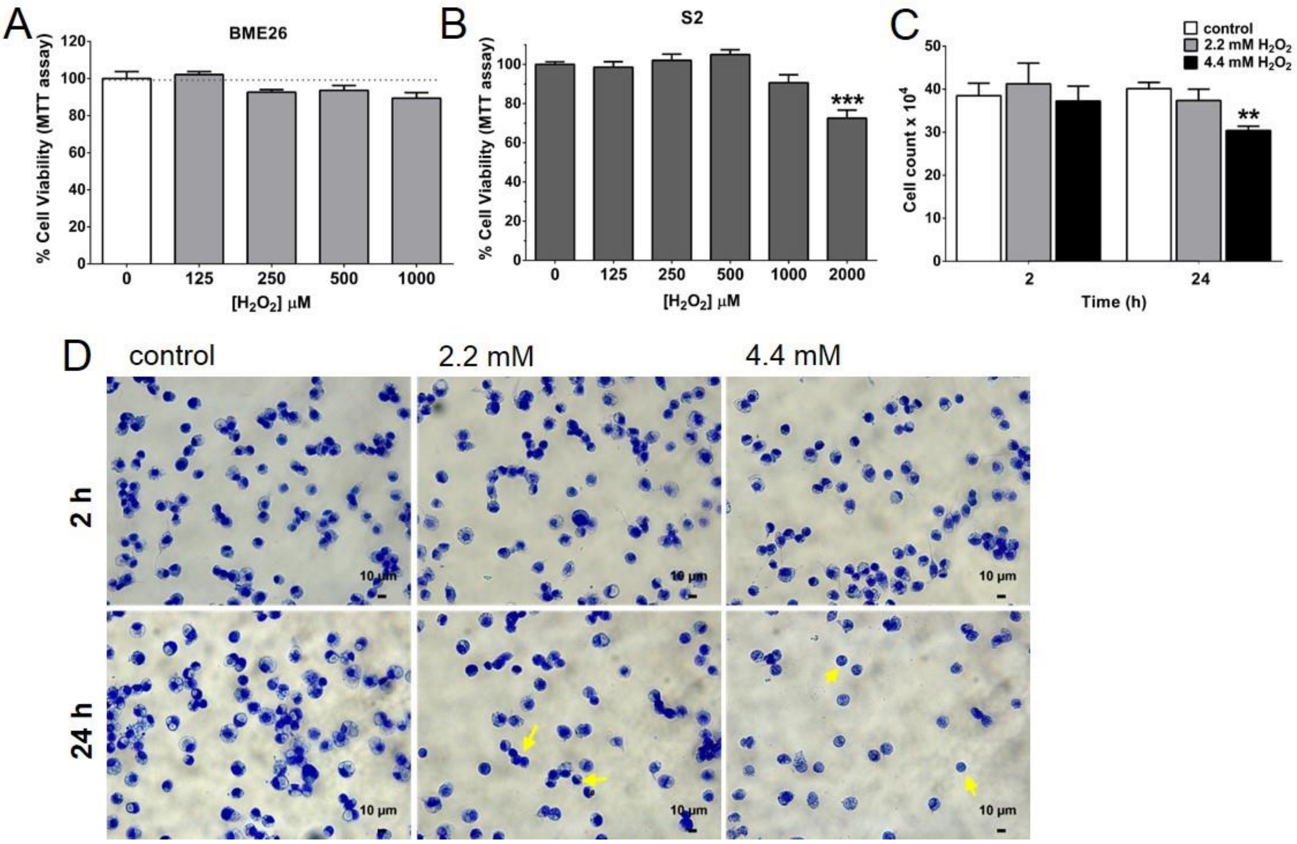
A
BME26
% Cell Viability (MTT assay)
0
125
250
500
1000
[H_2O_2] μM
B
S2
% Cell Viability (MTT assay)
***
0
125
250
500
1000
2000
[H_2O_2] μM
C
control
2.2 mM H_2O_2
4.4 mM H_2O_2
Cell count x 10^4
**
2
24
Time (h)
D
control
2.2 mM
4.4 mM
2 h
24 h
10 μm

Figure S2

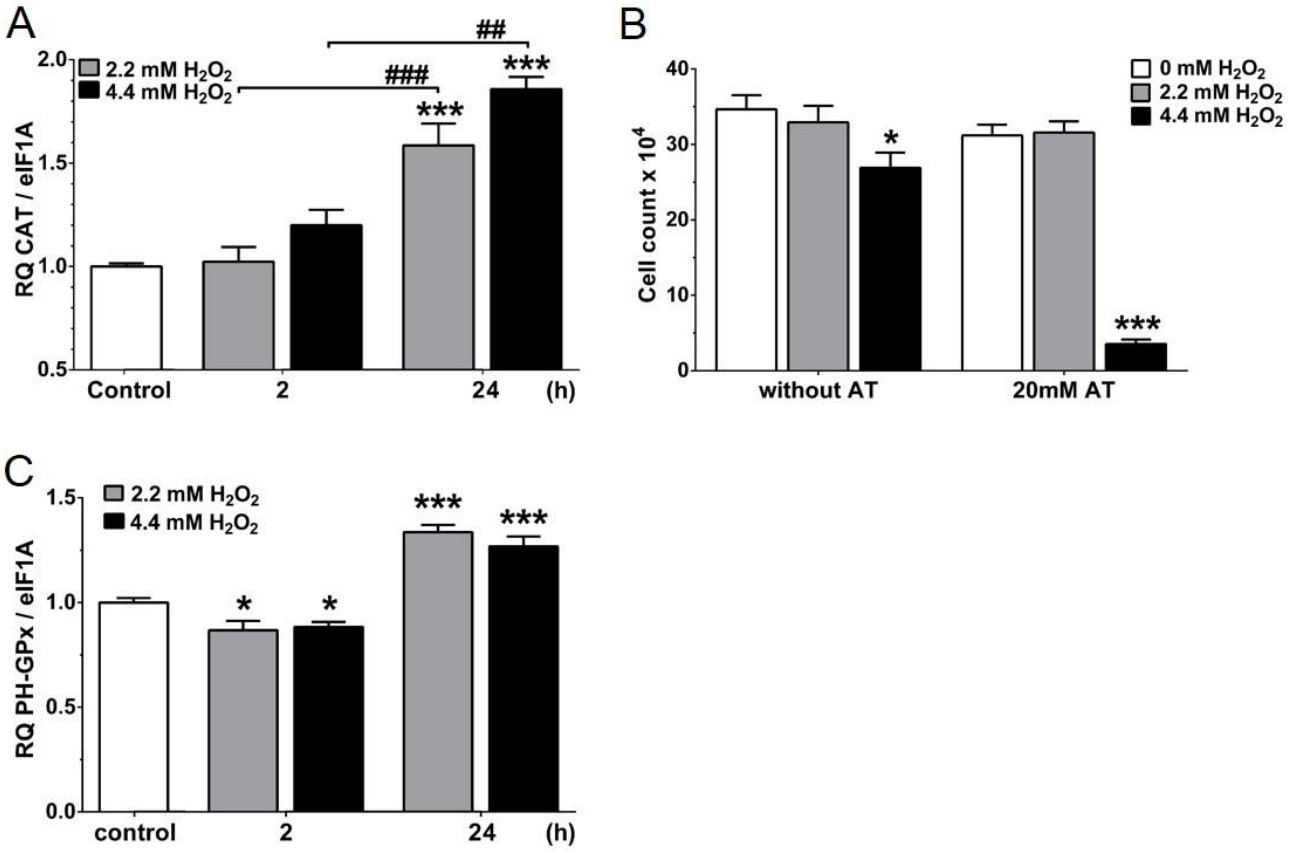
A
2.2 mM H2O2
4.4 mM H2O2
##
###
***
***
RQ CAT / eIF1A
2.0
1.5
1.0
0.5
Control
2
24
(h)
B
0 mM H2O2
2.2 mM H2O2
4.4 mM H2O2
*
***
Cell count x 10^4
40
30
20
10
0
without AT
20mM AT
C
2.2 mM H2O2
4.4 mM H2O2
***
***
*
*
RQ PH-GPx / eIF1A
1.5
1.0
0.5
0.0
control
2
24
(h)

Figure S3

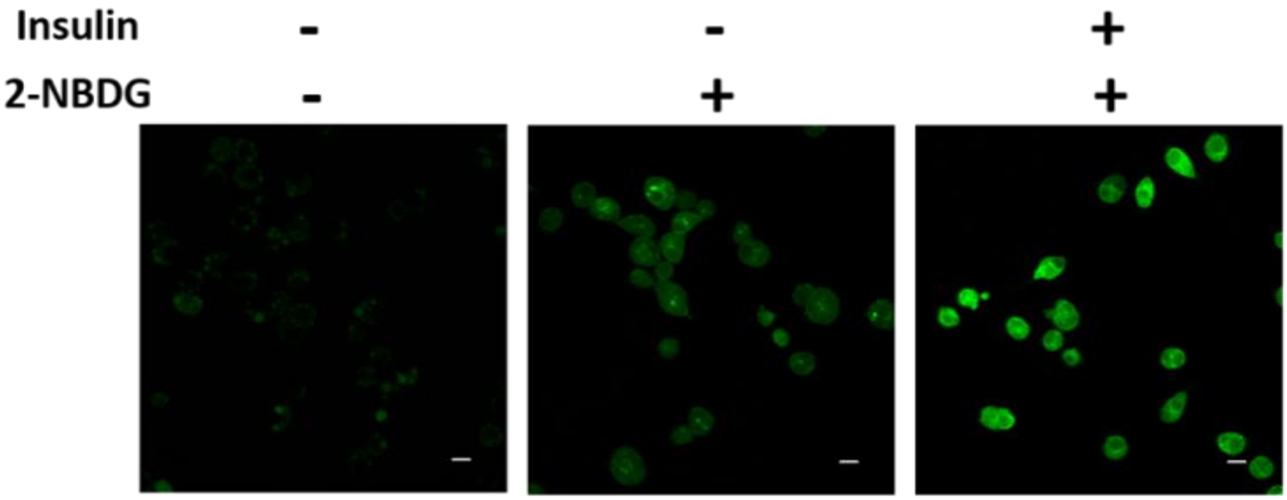

Figure S4

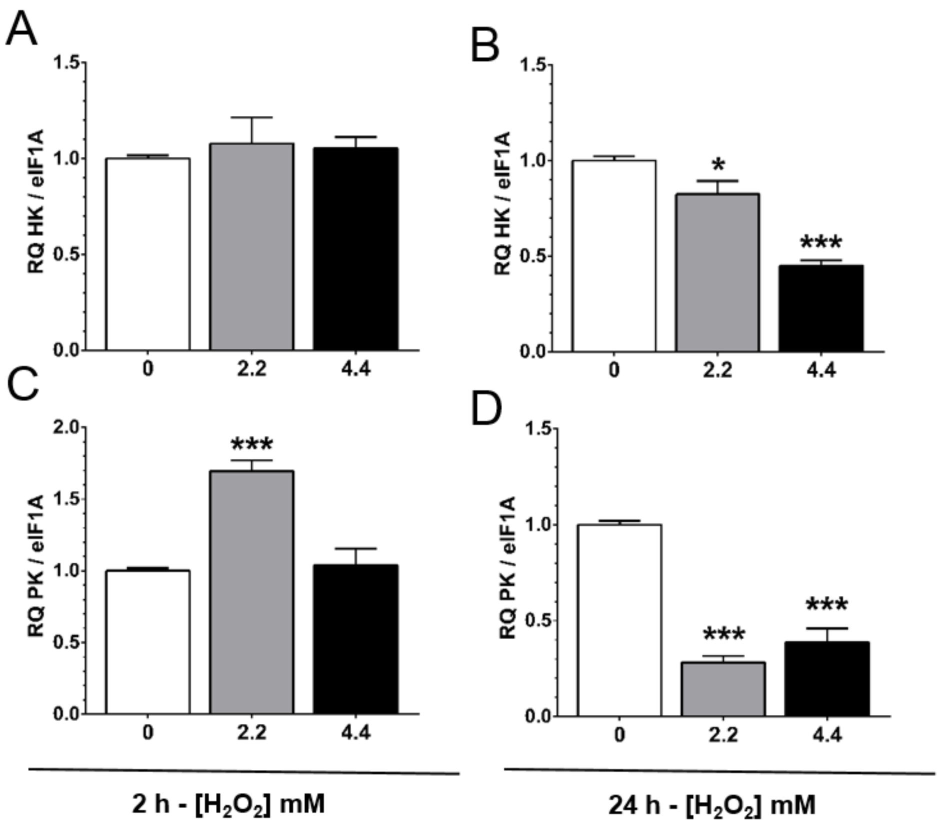
A
B
C
D
RQ HK / eIF1A
RQ PK / eIF1A
1.5
1.0
0.5
0.0
2.0
0
2.2
4.4
*
***
2 h - [H_2O_2] mM
24 h - [H_2O_2] mM

Figure S5

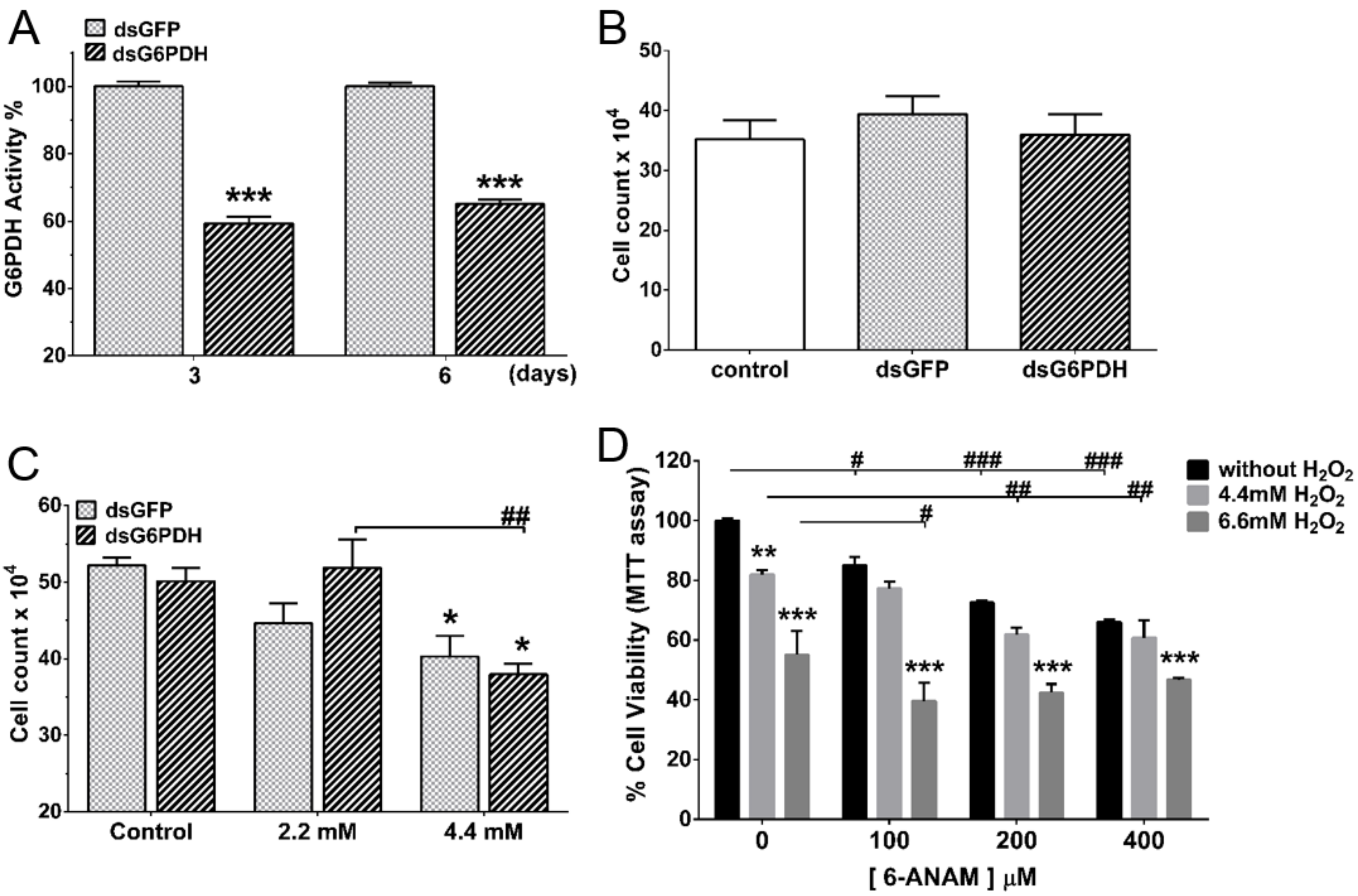

A
dsGFP
dsG6PDH
G6PDH Activity %
***
***
3
6
(days)
B
Cell count x 10^4
control
dsGFP
dsG6PDH
C
dsGFP
dsG6PDH
Cell count x 10^4
##
*
*
Control
2.2 mM
4.4 mM
D
without H_2O_2
4.4mM H_2O_2
6.6mM H_2O_2
% Cell Viability (MTT assay)
#
###
###
##
##
#
**
***
***
***
***
[6-ANAM] μM

Supplemental Methods

RNA Extraction and Relative Quantification (RQ) of Gene Transcription by Real-Time PCR

Total RNA was extracted from BME26 cells harvested from 24-well plates using Trizol reagent (Invitrogen, Grand Island, NY, USA) according to the manufacturer's instructions. One microgram of total RNA was reverse transcribed using the High-Capacity cDNA Reverse Transcription-M-MLV kit (Takara Biotechnology, Shiga, Japan). Relative transcription was analyzed with cDNA template in a quantitative PCR using the LightCycler 480 II platform (Roche, Mannheim, Germany) and the primers described in Table 1. Serial dilutions of the cDNA were used to construct a calibration curve. Reaction efficiencies between 85% and 100% were determined from calibration curves for each set of primers in 10 µL reactions. The *R. microplus* elongation factor-alpha gene (Elf1A) was used as reference [1] to normalize the reactions. cDNA from control, untreated BME26 cells was used as a calibrator for the assays. The relative expression of the calibrators was assigned a value of 1 unit. Statistical analyses (mean and standard deviation) were performed on data from three independent experiments. Relative expression was determined using the Cp values from each run in Relative Expression Software Tool [2].

For confirmation of G6PDH and NADP-ICDH knockdown by RT-qPCR, 1 µg total RNA was reverse transcribed with the High Capacity cDNA Reverse transcription kit (Invitrogen, USA). Relative transcription was analyzed using cDNA as a template for quantitative PCR in the StepOne Plus platform (Applied Biosciences). Gene sequences for $NADP^+$-dependent isocitrate dehydrogenase (NADP-ICDH) and catalase (CAT) from different species were retrieved from the GenBank database and used to conduct a BLAST search of an *R. microplus* transcriptome database generated using the Illumina Solexa sequencing platform (BioProject ID PRJNA232001 at Transcriptome Shotgun Assembly (TSA) database, GenBank). The *R. microplus* gene sequences for hexokinase (HK), pyruvate kinase (PK), glucose-6-phosphate dehydrogenase (G6PDH) and phospholipid-hydroperoxide glutathione peroxidase (PH-GPx) were obtained from GenBank. Specific primers for the genes were designed based on these *R. microplus* sequences (see Table 1).

Double-Stranded RNA (dsRNA) Synthesis and Delivery into BME26 Cells

Oligonucleotide primers containing T7 promoter sequence were synthesized for the *in vitro* transcription and synthesis of dsRNA using RiboMAX™ Express RNAi System Kit (Promega, Madison, WI, USA). cDNA from BME26 cells was used for dsRNA synthesis, using the primers listed in Table1. The dsRNA was purified according to the manufacturer's instructions, and its concentration was measured at 260 nm. An aliquot of dsRNA was analyzed by agarose gel electrophoresis to check for degradation. An unrelated dsRNA for GFP was used as negative control for the RNAi-induced gene silencing experiment. The size of the synthesized G6PDH and NADP-ICDH dsRNA complex were 553 bp and 565 bp, respectively. Double-stranded RNA was applied as described previously [3].

BME26 cells were seeded in 24-well plates, and 4 μg of dsRNA were added in 200 μL of medium per well by gently mixing. After 24-h incubation, 300 μL of complete medium were added to each well, and plates were further incubated at 34 °C for 48 h. At this point (3 days after dsRNA addition) the cells were collected for assays. G6PDH gene silencing was confirmed by RT-qPCR, as well as by enzyme activity assay. To verify the persistence of G6PDH gene silencing after the initial 3 days, culture medium was fully replaced by 500 μL of complete medium and incubated for additional 72 h. Cells were then collected to analyze G6PDH enzymatic activity (prolonged exposure assay, 6 days after dsRNA addition) [3]. For H_2O_2 challenge, BME26 cells (3 days after dsRNA addition) had their medium replaced (500 μL per well) and were treated with a single H_2O_2 bolus addition of 2.2 mM or 4.4 mM. Cell viability was measured after 24 h of incubation.

Phalloidin-Texas Red and Hoechst 33342 staining

After treatment, BME26 cells on round coverslips in a 24-well plate were washed twice with PBS pH 7.0 and fixed in 4% paraformaldehyde solution for 20 minutes at room temperature. After 2 washes, cells were incubated with absolute ice-cold acetone (stored at ≤–20°C) for 5 min, and then washed five times with PBS pH 7.0. Cells were incubated with a freshly prepared solution of 165 nM Texas Red Phalloidin (Molecular Probes) in the dark at 34 ^{o}C for 20 minutes, followed by a 5-min incubation with 25 μg/mL Hoestch 33342 (Aldrich Chem) at

room temperature. After being washed twice with PBS pH 7.0, the coverslips were prepared on slides with glycerol to be observed. The images were made using the Zeiss LSM710 confocal laser scanning microscope, using 40x magnification (Water Plan-Apochromat 40x / 1.0 DIC), through the program ZEN 2.3 (black edition).

Cell Proliferation Marker Ki67

After treatment, BME26 cells on round coverslips in a 24-well plate were washed twice with PBS pH 7.0 and fixed in 4% paraformaldehyde solution for 20 minutes at room temperature, permeabilized with absolute ice-cold acetone (stored at ≤–20°C) for 5 min, and washed five times with PBS pH 7.0. Cells were incubated for 1 hour with 1% BSA in PBS pH 7.0 (blocking solution), followed by an overnight incubation with Ki67 antibody (Abcam, anti-Ki67 antibody, #ab15580) diluted 1:200 in blocking solution. After that, cells were washed 3 times, incubated for 2 h with Anti-rabbit IgG secondary antibody conjugate-Alexa Fluor 555 (Cell Signaling, # 4413) diluted 1:1000 in blocking solution, followed by a 15-min incubation with DAPI (1 µg/mL). Cover slips were mounted in glycerol after 2 washes, and observed by microscopy. Image acquisition was performed using the Zeiss LSM710 confocal laser scanning microscope, with 40x magnification (Water Plan-Apochromat 40x / 1.0 DIC), through the program ZEN 2.3 (black edition). Cells were counted using cell counter Manual Counting plugin on ImageJ software [4]. Among the nuclei labeled with anti-Ki67, different degrees of intensity were identified corresponding to variation in Ki67 protein levels. Two categories (soft and bright) of red nuclei were separately normalized to total nuclei (DAPI stained) as 100%.

Measurement of Cellular ROS Level

After treatment, BME26 cells under round coverslips in a 24-well plate were washed twice with PBS pH 7.0 and incubated for 15 minutes with 300 µL of 50 µM DHE (Dihydroethidium; Sigma-Aldrich, #D7008) solution in PBS in the dark under gentle shaking. Then, cells were fixed with 4% paraformaldehyde for 20 min. After washed twice with PBS pH 7.0, coverslips were prepared on slides with glycerol to be immediately observed by microscopy. Image acquisition was performed using the Zeiss LSM710 confocal laser scanning microscope, using

40x magnification (Water Plan-Apochromat 40x / 1.0 DIC), through the program ZEN 2.3 (black edition). The quantification of mean fluorescence intensity (F.I.) per cell area in mm^2 was calculated by ZEN 2.3 software (Blue edition), from Carl Zeiss Microscopy GmbH, 2011.

Mitochondrial superoxide indicator for live cell images MitoSOX-Red

The Mitosox fluorescence probe (Molecular probes, invitrogen, # M36008) was used to verify the generation of mitochondrial superoxide in living cells, the entire procedure was performed according to the manufacturer's protocol. After treatment, adherent cells on the plate cover (Petri dishes for confocal microscopy) were washed twice with 500 µL of HBSS solution (prepared as recommended by the manufacturer), and then incubated with 500 µL of 5 µM Mitosox, diluted in HBSS solution, for 10 minutes at 34 °C in the dark. After three washes with HBSS solution, the cells were observed by confocal microscopy. Image acquisition was performed using the Zeiss LSM710 confocal laser scanning microscope, using 63x magnification (LD plan-neofluar 63x / 1.0 DIC), through the software ZEN 2.3 (black edition).

Glucose Uptake Assay

D-glucose fluorescent analog, 2-NBDG (2-[N-(7-nitrobenz-2-oxa-1,3-diazol-4-yl)amino]-2-deoxy-d-glucose) (Molecular probes, #N13195) was used as an indicator for measuring glucose uptake by the BME26 cells after H_2O_2 treatment [5]. After 2 h incubation with 2.2 mM or 4.4 mM H_2O_2, adherent cells on the plate cover (Petri dishes for confocal microscopy) were washed twice with 500 µL of L15 medium without glucose supplementation [6]. 2-NBDG glucose analog (100 µM final concentration) was added to 500 µL medium and incubated for 15 minutes at 34 °C in the dark. After two washes with L15 medium without glucose supplementation, the cells were observed in confocal microscopy. An additional positive control of glucose uptake 2-NBDG assay was made using insulin, according to previous studies [3,7]. BME26 cells were incubated for 1 h in the presence or absence of 1 mM insulin in L15 medium (without glucose) with 100 µM 2-NBDG. Image acquisition was performed using the Zeiss LSM710 confocal laser scanning microscope, at 40x magnification (Water Plan-Apochromat 40x / 1.0 DIC), through the software ZEN 2.3 (black edition). The

quantification of mean fluorescence intensity (F.I.) per cell area in mm^2 was calculated by ZEN 2.3 software (Blue edition), from Carl Zeiss Microscopy GmbH, 2011.

Oxygen Consumption

Treatment with H_2O_2 was done directly on the cell culture bottle and incubated for different times at 34 °C. After treatment, the medium was discarded, the cells were washed twice with 2 mL PBS pH 7.0, and resuspended in 2 ml PBS pH 7.0. Cells were counted in hemocytometer chamber [8] and 10^7 cells in 1 mL of PBS were added into Oxygraph cuvette. Total oxygen consumed by BME26 cells after H_2O_2 treatment, measured for 10 minutes, was determined using a Clark-type electrode (by Oxytherm liquid phase electrode, Hansatech Instruments Ltd). The calibration process was performed using complete air-saturated buffer at 28 °C as 100%. Measurements were carried out in 1.5 mL PBS pH 7.0 and the rate of oxygen consumption was calculated in µmol O_2 / min / 10^7 cells. A solution containing 1 mM KCN to inhibit cytochrome oxidase was used as negative control to confirm cellular respiration. Three assays were performed using 1×10^7 cells /mL for three independent experiments in triplicate.

NADPH Determination

Samples were prepared as described above for enzymatic activity. The cell lysate was assayed for NADPH determination using the NADP/NADPH Quantification Kit (Sigma Aldrich). NADPH levels in samples was determined spectrophotometrically by monitoring absorbance at 450 nm according to the manufacturer’s protocol.

Panotic staining

Panotic staining was done according to manufacturer’s instructions (Laborclin Brazil, #620529). Cells were incubated for 1 minute in solution 1 (0.1% triarylmethane) and 1 minute in solution 2 (0.1% xanthenes). After 2 washes with distilled water, cells were incubated for 3 minutes in solution 3 (0.1% thiazines) and washed 3 times in distilled water. Cover slides were prepared in glycerol and observed under microscope. Bright field microscopy images were captured using the Axio Scope.A1 polarized light microscope, Zeiss, through the Blue Zeiss

software.

G6PDH and Catalase chemical inhibition

BME26 cells were treated with 100 µM, 200 µM or 400 µM of 6-ANAM (6-aminonicotinamide, Sigma-Aldrich, #A68203) for 24 h. Then, we performed a single H_2O_2 bolus addition (4.4 mM or 6.6 mM) and the samples were incubated for additional 24 h, after which cell viability by MTT assay was assessed. BME26 cells were treated with catalase inhibitor, 3-amino-1,2,4-triazole (AT, Sigma No. A8056), at $2x10^{-2}$ M for 4 h, then we performed a single H_2O_2 bolus addition (at 2.2 mM and 4.4 mM) incubated for 24 h, after which cell viability was determined using a Neubauer hemocytometer with trypan blue exclusion technique.

REFERENCES

1. Nijhof, A. M., Balk, J. A., Postigo, M. & Jongejan, F. Selection of reference genes for quantitative RT-PCR studies in Rhipicephalus (Boophilus) microplus and Rhipicephalus appendiculatus ticks and determination of the expression profile of Bm86. *BMC Mol. Biol.* **10,** (2009).
2. Pfaffl, M. W. A new mathematical model for relative quantification in real-time RT-PCR. *Nucleic Acids Res.* **29,** 45e–45 (2001).
3. de Abreu, L. A. *et al.* The conserved role of the AKT/GSK3 axis in cell survival and glycogen metabolism in Rhipicephalus (Boophilus) microplus embryo tick cell line BME26. *Biochim. Biophys. Acta - Gen. Subj.* **1830,** 2574–2582 (2013).
4. T. Ferreira, W. R. *ImageJ User Guide IJ 1.46r*. *IJ 1.46r* (2012). doi:10.1038/nmeth.2019
5. Zou, C., Wang, Y. & Shen, Z. 2-NBDG as a fluorescent indicator for direct glucose uptake measurement. *J. Biochem. Biophys. Methods* **64,** 207–215 (2005).
6. Munderloh, U. G. & Kurtti, T. J. Formulation of medium for tick cell culture. *Exp. Appl. Acarol.* **7,** 219–229 (1989).
7. Abreu, L. A. de *et al.* Exogenous insulin stimulates glycogen accumulation in Rhipicephalus (Boophilus) microplus embryo cell line BME26 via PI3K/AKT pathway. *Comp. Biochem. Physiol. - B Biochem. Mol. Biol.* **153,** 185–190 (2009).
8. Cadena-Herrera, D. *et al.* Validation of three viable-cell counting methods: Manual, semi-automated, and automated. *Biotechnol. Reports* **7,** 9–16 (2015).
